# In-Store Cosmetic Testers Harbour Resistant Bacteria: A Pilot Survey of Microbial Contamination and Antimicrobial Susceptibility in Portuguese Pharmacies

**DOI:** 10.3390/microorganisms14092120

**Published:** 2026-09-21

**Authors:** Sónia Ribeiro, Jéssica Duarte Silva, Patrícia Pacheco, Célia Fortuna Rodrigues

**Affiliations:** 1 Department of Pharmaceutical Sciences, University Institute of Health Sciences (IUCS-CESPU), 4585-116 Gandra, Portugal; a30643@alunos.cespu.pt (S.R.); patricia.pacheco@cespu.pt (P.P.); 2 Associate Laboratory i4HB—Institute for Health and Bioeconomy, CESPU University, 4585-116 Gandra, Portugal; a26946@alunos.cespu.pt; 3 UCIBIO—Research Unit on Applied Molecular Biosciences, Translational Toxicology Research Laboratory, CESPU University, 4585-116 Gandra, Portugal; 4 LEPABE/ALiCE—Laboratory for Process Engineering, Environment, Biotechnology and Energy and Associate Laboratory in Chemical Engineering, Faculty of Engineering, University of Porto, Rua Dr. Roberto Frias, 4200-465 Porto, Portugal

**Keywords:** cosmetic testers, community pharmacy, microbiological contamination, multidrug resistance, antimicrobial susceptibility, public health

## Abstract

Cosmetic testers in community pharmacies may act as a source of microbial transmission, but their microbiological safety remains poorly characterised. This study assessed microbial contamination of 42 cosmetic testers (eight product categories) from community pharmacies in northern Portugal using culture-based methods, identification and Kirby–Bauer disk diffusion. We characterised the antimicrobial susceptibility of the recovered isolates. Microbial growth was detected in 25/42 testers (59.5%), with polymicrobial contamination in 16.7% of samples. Out of the 25 positive growth products, 33 characterised bacterial isolates were yielded. *Staphylococcus* sp. predominated (22/33; 66.7%), followed by *Pseudomonas* sp. (4/33; 12.1%), *Streptococcus* sp. (3/33; 9.1%), *Acinetobacter* sp. (2/33; 6.1%) and *Bacillus* sp. (2/33; 6.1%). Among categories represented by more than one tester, anti-ageing products showed the highest proportion of microbial growth (7/8; 87.5%), followed by sunscreens (10/16; 62.5%). Overall, 12/33 bacterial isolates (36.4%) met the study-defined operational multidrug-resistance criterion, all of which were *Staphylococcus* sp. (12/22; 54.5%). These findings demonstrate that viable bacteria exhibiting phenotypic antimicrobial resistance can be recovered from in-use cosmetic testers in community pharmacies and support further investigation of their microbiological and antimicrobial-resistance characteristics. This reinforces that pharmacy cosmetic testers may harbour a diverse, and often multidrug-resistant, microbial population of clinical relevance, supporting the need for strengthened tester hygiene and control practices.

## 1. Introduction

According to the Food and Drug Administration (FDA) and EU Regulation No. 1223/2009, cosmetic products are substances or mixtures intended for application to the external parts of the human body. Although cosmetics are not required to be sterile, their microbiological quality must remain compatible with consumer health during intended and foreseeable use [1,2,3]. Nonetheless, it is regulated that the presence of microorganisms in a product cannot adversely affect consumer safety or the products’ quality (Table 1) [4].

Due to safety concerns, finished products are expected to comply with the microbiological limits summarised in Table 1 [4]. Certain species, such as *Staphylococcus* sp., may cause respiratory and skin infections, including desquamation and acne, and have also been associated with chronic wounds [5]. *Bacillus* sp. are typically transient skin microflora; however, *Bacillus cereus* contamination in eye cosmetics can lead to severe infections [6]. In the wider cosmetics literature, fungal contamination (yeasts and, in some product types, moulds such as *Aspergillus* and *Penicillium*) is also recognised as a quality concern [7].

Cosmetic testers, which allow consumers to sample products before purchase, represent an underrecognised microbiological concern: repeated handling by multiple users, often under uncontrolled hygiene conditions, may act as a route for microbial transmission via direct contact with skin, lips, or mucous membranes, particularly in pharmacy and health-associated retail settings where products may be accessed by individuals with compromised skin barriers or increased susceptibility to infection [8,9,10]. Previous surveys of used or in-store cosmetics have reported high contamination rates: 79–90% of used products in the UK [8], frequent isolation of *S. epidermidis* from unsealed makeup testers in Saudi Arabia [9], and antibiotic-resistant *Staphylococcus* and *Bacillus* from makeup testers in Algerian retailers [10]. Similar patterns have been reported outside Europe: shared cosmetic kits at beauty salons showed comparable bacterial and fungal contamination and associated metabolite exposure [11,12], and a recent consumer survey identified key pathogenic bacteria on lip cosmetic testers alongside low consumer awareness of the associated risk [13]. However, the available evidence remains methodologically heterogeneous, with substantial variation in sampling strategies, microbiological methods, product categories, and reporting approaches [14,15]. Most studies primarily report contamination prevalence and organism identification, while antimicrobial susceptibility profiles of the recovered isolates are less frequently characterised, leaving the clinical and microbiological significance of these isolates insufficiently understood.

It is known that damaged skin may not provide an adequate primary barrier, since it relies on multiple defence mechanisms [16,17]. Individuals with compromised skin integrity due to inflammatory dermatoses, allergies, burns, physical trauma, as well as those with immunosuppression, are more susceptible to infections from contaminated products [18]. The skin microbiome, including commensals such as *S. epidermidis*, plays a role in maintaining skin health and preventing pathogen overgrowth; disruption of this balance (dysbiosis) has been linked to several dermatological conditions [5,19]. Contaminated products can lead to infections, allergic reactions, and skin irritations, highlighting the importance of monitoring and controlling microbial contamination to protect consumers and maintain industry credibility [8,14]. Although microbial contamination of used and in-store cosmetic products has been documented in several settings, the available studies differ substantially in the products examined, sampling approaches, microbiological methods and level of microbial characterisation [8,9,10,11,12,13]. Most studies have focused primarily on detecting contamination and identifying the microorganisms recovered, whereas antimicrobial susceptibility has been investigated in comparatively few studies and has often been restricted to selected bacterial groups or isolates [10]. Consequently, there remains limited information on the antimicrobial-resistance profiles of the broader bacterial populations recovered from shared cosmetic testers. This gap is particularly relevant in healthcare-associated retail environments. To our knowledge, microbial contamination, together with antimicrobial susceptibility of bacteria recovered from multi-user cosmetic testers, has not previously been characterised in Portuguese community pharmacies.

In addition, customised “made-on-the-spot” cosmetics present additional risks, as preservation cannot be guaranteed, and their production may lead to cross-contamination from the environment, utensils, or handlers [2]. The presence of antibiotic-resistant microorganisms in cosmetic products is a growing concern, as contaminated products and their manufacturing environments can act as reservoirs of resistant organisms [20,21].

Resistant biofilms have been documented in cosmetics-associated environments, illustrating a plausible route by which contaminated production or retail environments could increase the risk of hard-to-treat exposures, particularly for individuals with compromised skin or immunosuppression [20]. The global rise in antibiotic consumption and resistance parallels concerns about preservatives in cosmetics, which may select for resistant strains [20,21]. Proper preservation, through traditional or natural antimicrobial agents, ensures product safety without compromising skin health [22,23].

Although consumer-level risk is low due to effective concentrations in finished products, poor manufacturing practices and inadequate microbiological control in production environments may facilitate microbial resistance, emphasising the importance of good manufacturing practices, preservation, and continuous monitoring [21,22,24]. Within a One Health framework, community-level reservoirs of multidrug-resistant organisms—such as widely handled retail products—are increasingly recognised as contributors to antimicrobial resistance beyond clinical settings. Although finished cosmetic products are subject to defined microbiological limits, no previous study has characterised microbial contamination and antimicrobial resistance in in-use, multi-user testers from Portuguese community pharmacies. Community pharmacies were selected because they are a healthcare-adjacent retail setting in which consumers may reasonably expect higher hygiene standards than in general cosmetics stores; sampling was restricted to pharmacies in northern Portugal and is therefore a defined scope rather than a comparison across retail types.

We hypothesised that in-use cosmetic testers available in community pharmacies would yield recoverable microbial contamination, including bacterial isolates exhibiting phenotypic antimicrobial resistance. Therefore, this pilot study aimed to characterise microbial contamination in cosmetic testers collected from community pharmacies in northern Portugal, identify the recovered microorganisms, describe their antimicrobial susceptibility profiles, and explore differences in microbial recovery across product categories.

## 2. Materials and Methods

### 2.1. Samples

Pharmacies were selected by convenience within the study region; within each participating pharmacy, all cosmetic testers available for public use at the time of visit were included, covering eight product categories. A total of 42 cosmetic testers were collected between November and December 2025 from community pharmacies located in the municipalities of Guimarães and Vila Nova de Famalicão, Portugal. Samples were identified according to their National Product Code (“Código Nacional de Produto”, CNP). Approximately 3 mL of each product was aseptically transferred into sterile tubes and stored at 4 °C until microbiological analysis. All microbiological procedures were performed using sterile materials and aseptic techniques.

### 2.2. Microbiological Growth

For microbiological analysis, samples were directly inoculated onto Blood Agar (BA), Nutrient Agar (NA), Sabouraud Dextrose Agar (SDA), MacConkey Agar and Mannitol Salt Agar (MSA) (Merck, Darmstadt, Germany). The inoculated media were incubated under appropriate growth conditions. Blood Agar plates were incubated at 37 °C in an atmosphere containing 5% CO_2_, while the remaining media were incubated aerobically at 37 °C. Prior to inoculation across the five media types, after the products were homogenised by vortexing, an aliquot of around 0.1 mL was first inoculated onto an all-purpose, non-selective medium; colonies obtained from this initial growth were then subcultured onto the selective/differential media, as described above. Bacterial cultures were examined after 24–48 h, whereas SDA plates were monitored for up to 5 days. Colonies presenting distinct morphological characteristics were selected and subcultured to obtain pure isolates. Uninoculated media plates from each batch were incubated in parallel as sterility controls.

### 2.3. Microbiological Identification

Preliminary characterisation included observation of colony morphology and Gram staining. Isolates were further subjected to conventional biochemical testing, including catalase, coagulase, oxidase, indole, urease, citrate utilisation, motility, hydrogen sulfide production, bile esculin hydrolysis, growth in 6.5% NaCl, mannitol fermentation, Methyl Red (MR), Voges–Proskauer (VP), oxidative-fermentative (OF) metabolism and Kligler Iron Agar reactions. Additional differentiation tests using novobiocin, optochin, bacitracin and trimethoprim–sulfamethoxazole (SXT) discs were performed when required (bioMérieux, Marcy-l’Étoile, France). Species identification was confirmed using API identification systems (bioMérieux, Marcy-l’Étoile, France) according to the manufacturer’s instructions, and depending on the isolate primary identification.

### 2.4. Antimicrobial Susceptibility

After identification, fresh cultures were prepared for antimicrobial susceptibility testing. Susceptibility profiles were determined by the Kirby–Bauer disk diffusion method on Mueller–Hinton agar (Merck, Darmstadt, Germany). Bacterial suspensions were adjusted to a turbidity equivalent to a 0.5 McFarland standard before inoculation. Antibiotic panels were selected according to the genus identified. For Gram-negative non-fermenters (*Pseudomonas* sp., *Acinetobacter* sp.), the panel included imipenem, ciprofloxacin, and amikacin, with aztreonam additionally tested for *Pseudomonas* sp. and gentamicin and trimethoprim–sulfamethoxazole additionally tested for *Acinetobacter*. Colistin and polymyxin B were not included in this panel, as polymyxin susceptibility cannot be reliably determined by disk diffusion, since these large cationic molecules diffuse poorly through agar. For *Staphylococcus* sp., the panel included cefoxitin (as a screening marker for methicillin resistance/MRSA), penicillin, gentamicin, amikacin, ciprofloxacin, clindamycin, tetracycline, and trimethoprim–sulfamethoxazole. Linezolid and daptomycin, though clinically important for confirmed MRSA or VRE, were not included: these are reserved, last-line agents not typically part of first-line phenotypic screening panels for environmental or retail surveillance isolates, and daptomycin activity is calcium-dependent and cannot be reliably assessed by disk diffusion, requiring broth microdilution instead. The present panel was therefore designed for phenotypic MRSA screening (via cefoxitin) rather than as a comprehensive treatment-selection panel. For *Streptococcus* sp., the panel included vancomycin, tetracycline, trimethoprim–sulfamethoxazole, and optochin (used as a presumptive identification aid for *S. pneumoniae* rather than a susceptibility marker). Inhibition zone diameters were measured and interpreted according to the EUCAST clinical breakpoint tables, Version 16.0 (2026) [15], and isolates were classified as susceptible (S), susceptible with increased exposure (I), or resistant (R). *Bacillus* sp. isolates do not have genus-specific EUCAST clinical breakpoints; results for these isolates (cefoxitin, gentamicin, ciprofloxacin, clindamycin, tetracycline, trimethoprim–sulfamethoxazole) are therefore reported descriptively as a qualitative susceptibility profile rather than as formal EUCAST susceptible/intermediate/resistant categories, following current practice for genera without established breakpoints [15]. Multidrug resistance (MDR) was defined as acquired non-susceptibility to ≥1 agent in ≥3 antimicrobial categories, according to Magiorakos et al. [25]. As an internal consistency check, any tester initially recorded as “no growth” but subsequently found to show growth on re-inspection was re-incubated and re-assessed before assigning a final result (this occurred for two testers in this dataset); isolates from testers with multiple repeated plates were likewise re-incubated to confirm colony morphology before proceeding to identification and susceptibility testing.

Antimicrobial susceptibility testing was performed using genus-specific antibiotic panels. For *Pseudomonas* sp., four antibiotics representing four antimicrobial classes were tested: imipenem (carbapenem), aztreonam (monobactam), ciprofloxacin (fluoroquinolone), and amikacin (aminoglycoside). For *Acinetobacter* sp*.,* five antibiotics from four antimicrobial classes/groups were evaluated: imipenem (carbapenem), ciprofloxacin (fluoroquinolone), amikacin and gentamicin (aminoglycosides), and trimethoprim–sulfamethoxazole. For *Staphylococcus* sp., eight antibiotics representing seven antimicrobial classes/groups were tested: penicillin, cefoxitin, ciprofloxacin, amikacin, gentamicin, clindamycin, tetracycline, and trimethoprim–sulfamethoxazole. For *Streptococcus* sp., three antibiotics from three antimicrobial classes/groups were assessed: vancomycin, tetracycline, and trimethoprim–sulfamethoxazole. Finally, for *Bacillus* sp., four antibiotics representing four antimicrobial classes/groups were tested: ciprofloxacin, vancomycin, clindamycin, and tetracycline. The antimicrobial agents included in the susceptibility testing were selected according to their availability in the laboratory and their applicability to the respective bacterial genera based on EUCAST interpretative criteria. Consequently, the number and composition of antimicrobial classes tested varied among genera. Multidrug resistance (MDR) was therefore assessed using an operational definition adapted to the available antimicrobial panel. MDR operational definition is described in Section 2.6.

### 2.5. Statistical Analysis

Statistical analyses were performed using IBM SPSS Statistics, version 31.0.1.0 (IBM Corp., Armonk, NY, USA). The database comprised 60 analytical records from 42 cosmetic products, including 33 bacterial isolates (identified at the genus level and evaluated by antimicrobial susceptibility testing), four fungal isolates, six unidentified microorganisms, and 17 no-growth records. Product-level analyses (contamination, product class, multiple-isolate recovery) used the 42 cosmetic products as the analytical unit; antimicrobial susceptibility analyses used the 33 bacterial isolates.

For each isolate, a proportional resistance burden (R results/total antimicrobials tested) and a category-based non-susceptibility burden (non-susceptible categories/total categories tested) were calculated. Operational multidrug resistance (MDR) was defined according to Magiorakos et al. [25].

### 2.6. MDR Operational Definition

For the purposes of this study, multidrug resistance (MDR) was operationally defined at the genus level as resistance to at least one antimicrobial agent in three or more antimicrobial categories represented in the susceptibility-testing panel. This classification was applied consistently across all bacterial genera evaluated. Because the antimicrobial panels were limited to the agents tested in this study, the resulting MDR classification represents a panel-based phenotypic assessment and should not be interpreted as an application of species-specific MDR definitions.

Antimicrobial susceptibility testing was performed using the antimicrobial agents available for each bacterial group, and results were interpreted according to the applicable EUCAST clinical breakpoint criteria. MDR was defined as resistance to at least one microbial agent in three or more antimicrobial categories, following the framework proposed by Magiorakos et al. [25]. However, not all antimicrobial agents included in the MDR framework were available for testing. Consequently, MDR classification was based on the antimicrobial categories represented in the tested panel and should be interpreted within the scope of the antibiotics evaluated.

### 2.7. Product-Level Resistance Analysis

To account for the recovery of multiple *Staphylococcus* sp. isolates from the same cosmetic product, antimicrobial resistance was additionally analysed at the product level. Each cosmetic product was treated as one independent observational unit. For each antimicrobial class, a product was classified as resistance-positive when at least one Staphylococcus isolate recovered from that product exhibited acquired resistance to that class. Therefore, products yielding multiple Staphylococcus isolates contributed only once to the denominator for each antimicrobial class.

### 2.8. No Acquired Resistance Detection

For antimicrobial agents for which EUCAST provides resistance breakpoints in brackets, isolates with inhibition zones below the bracketed resistance breakpoint were classified as resistant (R), in accordance with EUCAST guidance. Because EUCAST advises against reporting isolates above these thresholds as susceptible or susceptible at increased exposure, such results were designated in this study as ‘no acquired resistance detected’ (NARD). NARD is a study-specific descriptive category indicating that phenotypic evidence of acquired resistance was not detected using the applicable EUCAST resistance threshold and should not be interpreted as equivalent to clinical susceptibility.

## 3. Results

### 3.1. Overall Contamination

**Figure 1:**
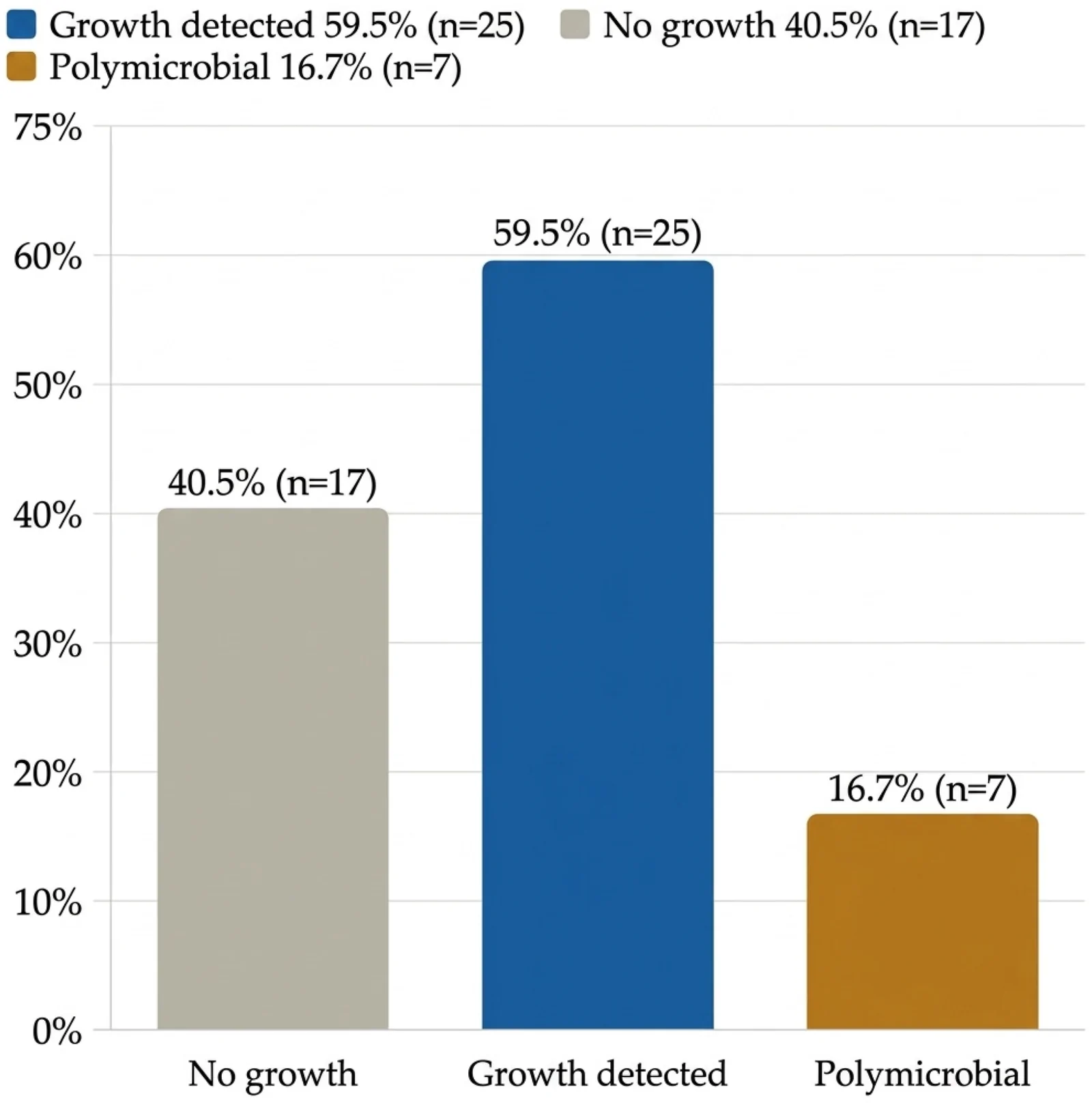
Prevalence of microbiological contamination across cosmetic testers (*n* = 42). Polymicrobial contamination (*n* = 7; 16.7%).

Of the 42 cosmetic testers sampled, microbial growth was detected in 25 (59.5%), while 17 (40.5%) showed no growth (Figure 1). Polymicrobial contamination, defined as the recovery of two or more distinct microbial isolates from the same cosmetic tester, was observed in 7/42 testers (16.7%).

### 3.2. Genus Distribution

**Figure 2:**
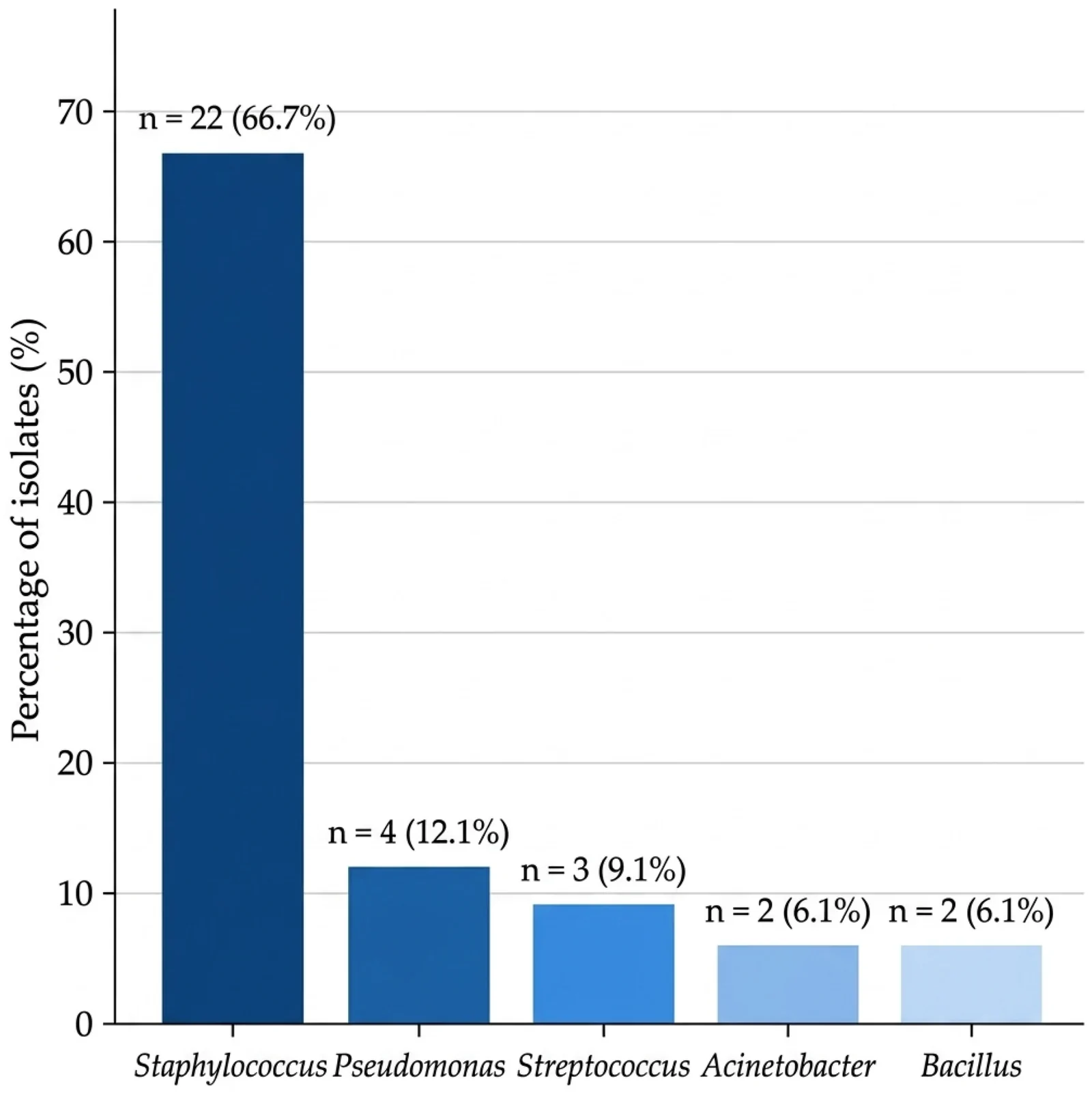
Relative frequency of bacterial genera among the 33 identified bacterial isolates. Bars represent the percentage of isolates; labels show *n* (%).

A total of 33 bacterial isolates were identified. *Staphylococcus* sp. was the predominant genus (*n* = 22; 66.7%), followed by *Pseudomonas* sp. (*n* = 4; 12.1%), *Streptococcus* sp. (*n* = 3; 9.1%), *Acinetobacter* sp. (*n* = 2; 6.1%), and *Bacillus* sp. (*n* = 2; 6.1%) (Figure 2).

**Figure 3:**
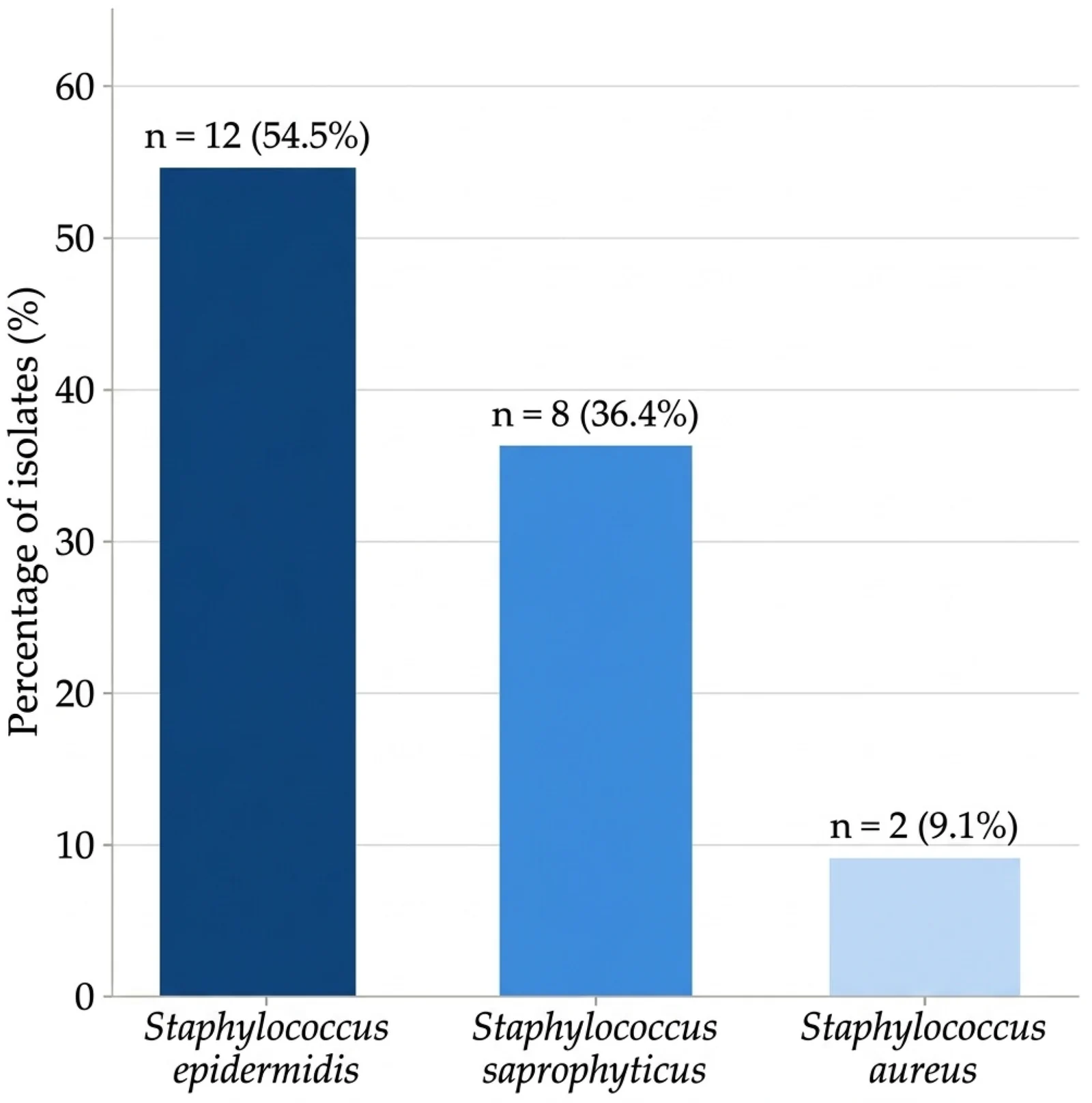
Species distribution among the Staphylococcus isolates (*n* = 22).

Among *Staphylococcus*, the *Staphylococcus epidermidis* was the most common, followed by *Staphylococcus saprophyticus* and *Staphylococcus aureus* (Figure 3). These species are not clinically equivalent: *S. aureus* is a well-recognised pathogen, whereas the coagulase-negative staphylococci (*S. epidermidis* and *S. saproph*y*ticus*) typically have lower baseline virulence as opportunistic skin commensals, but are relevant reservoirs of antimicrobial-resistance genes [26,27].

**Figure 4:**
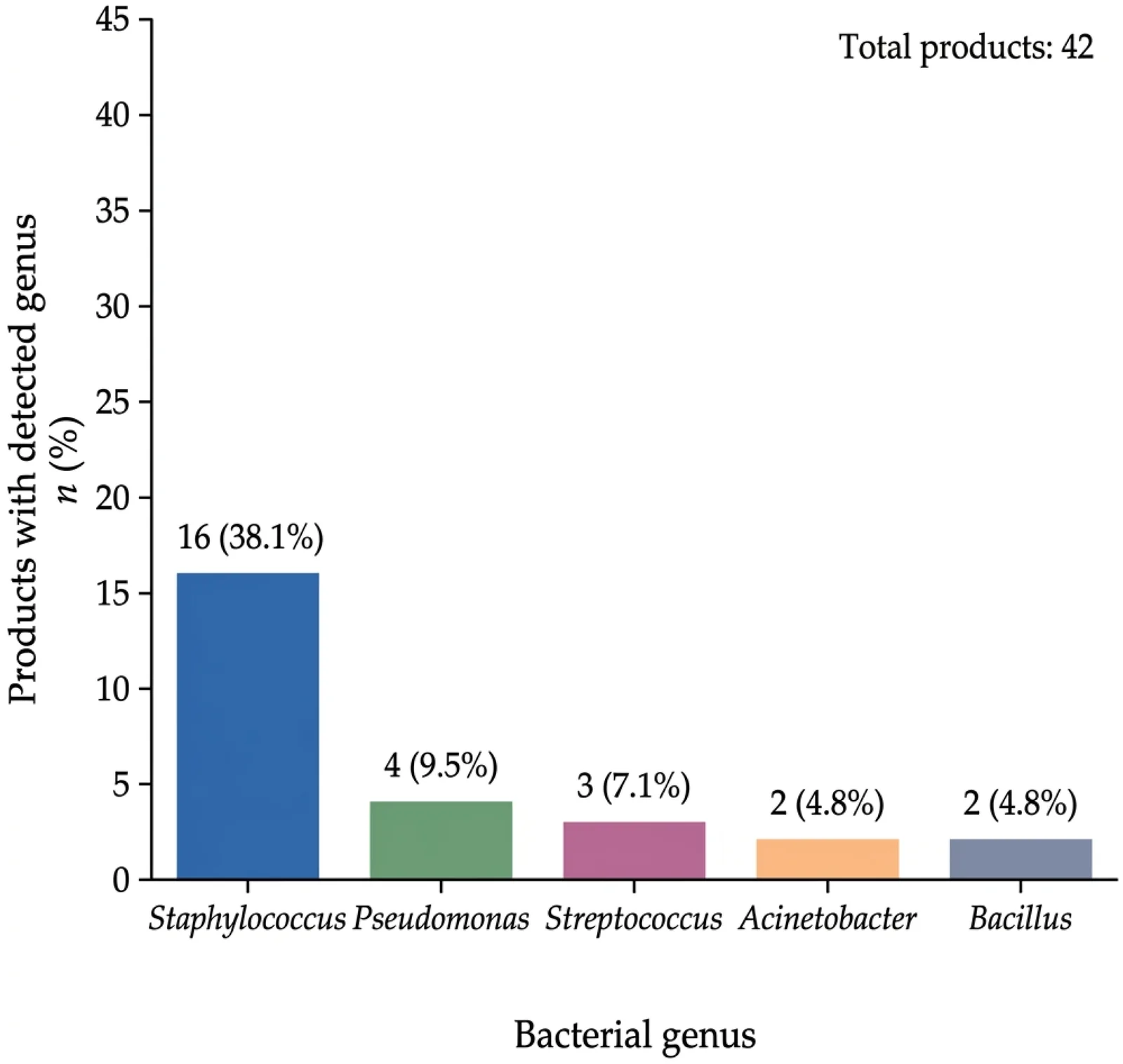
Product level genus distribution.

At the product level, *Staphylococcus* sp. recovered from 16/42 (38.1%) cosmetic products, *Pseudomonas* sp. from 4/42 (9.5%), *Streptococcus* sp. from 3/42 (7.1%), *Acinetobacter* sp. from 2/42 (4.8%), and *Bacillus* sp. from 2/42 (4.8%). Because more than one bacterial genus could be recovered from the same cosmetic product, these proportions are not mutually exclusive and were interpreted descriptively (Figure 4.)

### 3.3. Contamination by Category

**Figure 5:**
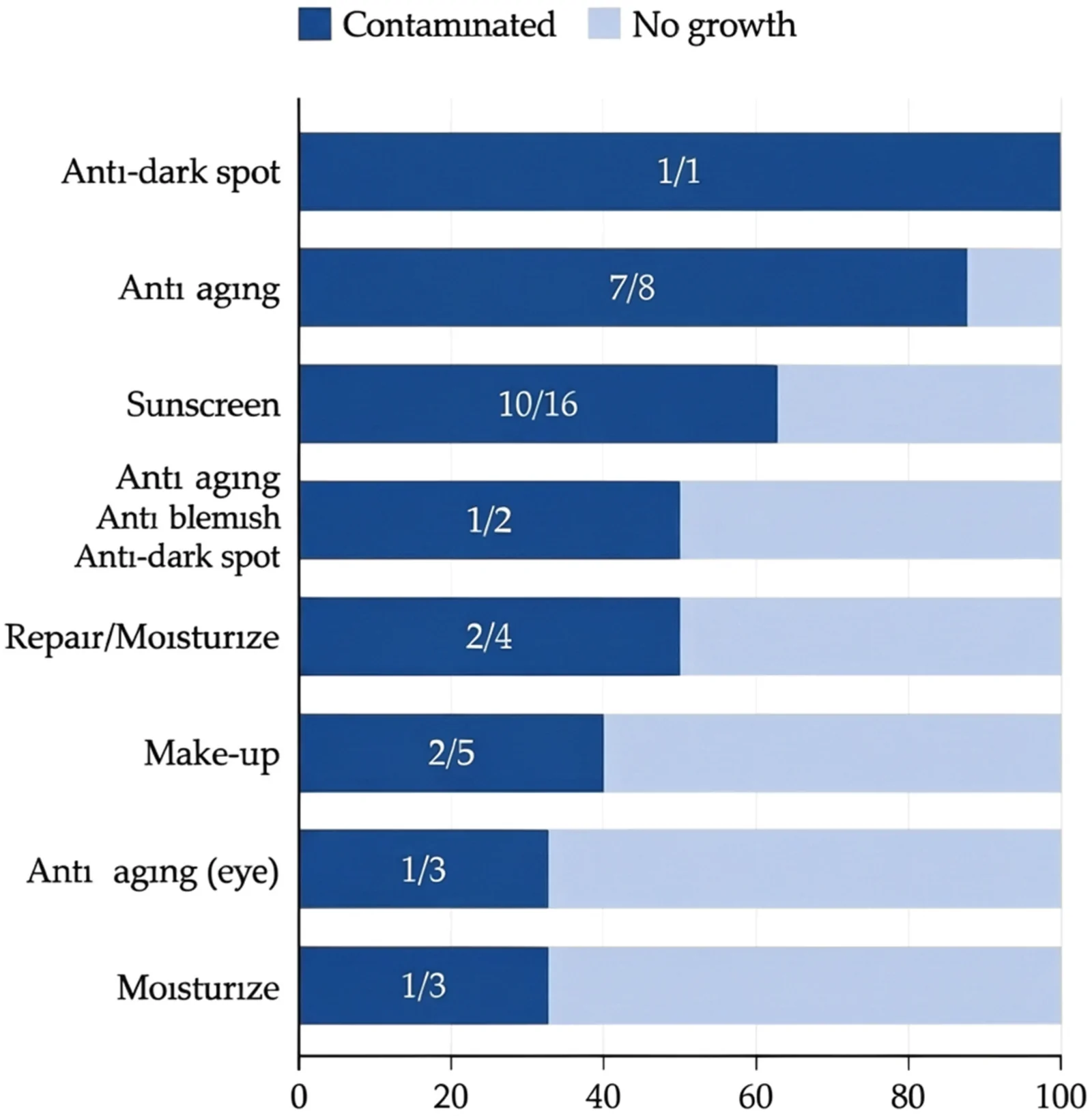
Proportion of contaminated testers by product category (*n* = 42). Numbers inside bars indicate absolute counts. ”No growth” should not be interpreted as absence of microbial contamination, as preservative neutralisation was not performed and residual preservative activity may have inhibited microbial recovery under the test conditions.

Contamination frequency varied across product classes, with the highest proportions observed in anti-dark-spot products (1/1; 100.0%) and anti-ageing products (7/8; 87.5%), followed by sunscreens (10/16; 62.5%). Intermediate contamination rates were observed for anti-ageing/anti-blemish/anti-dark-spot products (1/2; 50.0%) and repair/moisturiser products (2/4; 50.0%). Lower proportions were found in makeup products (2/5; 40.0%), anti-ageing eye products (1/3; 33.3%), and moisturisers (1/3; 33.3%) (Figure 5).

Anti-ageing products showed the highest contamination frequency (87.5%), followed by sunscreens, exhibiting a rate of contamination of 62.5%. These findings may reflect differences in formulation characteristics, water activity, packaging systems, frequency of manipulation, and cumulative exposure of testers to consumers, all of which can affect the opportunity for microbial introduction and persistence. Products such as anti-ageing creams and sunscreens are commonly formulated as emulsions containing an aqueous phase and are often repeatedly accessed during use, potentially increasing the relevance of preservative efficacy throughout the product’s in-use period. Nevertheless, the 100% contamination observed for anti-dark-spot products should be interpreted cautiously because this category was represented by only one product and therefore cannot be considered indicative of the broader product class. Overall, the observed variability supports further investigation of product-specific and use-related factors associated with contamination, rather than suggesting an intrinsic susceptibility of any particular cosmetic category (Figure 5).

### 3.4. MDR Profile

#### 3.4.1. Overall Antimicrobial Susceptibility Profile

Regarding antimicrobial resistance, Table 2 presents the sample identification and corresponding microorganism identified for each isolate, whereas Table 3, Table 4, Table 5, Table 6, Table 7 and Table 8 summarise the antimicrobial susceptibility profiles of the recovered isolates, including the specific antimicrobial panels tested and the resistance patterns observed for each genus.

Among the four *Pseudomonas* sp. isolates, resistance to aztreonam was observed in all isolates (4/4; 100%), while one isolate (1/4; 25%) additionally exhibited resistance to ciprofloxacin. None of the *Pseudomonas* sp. isolates met the criteria for an MDR phenotype based on the antimicrobial panel tested.

**Figure 6:**
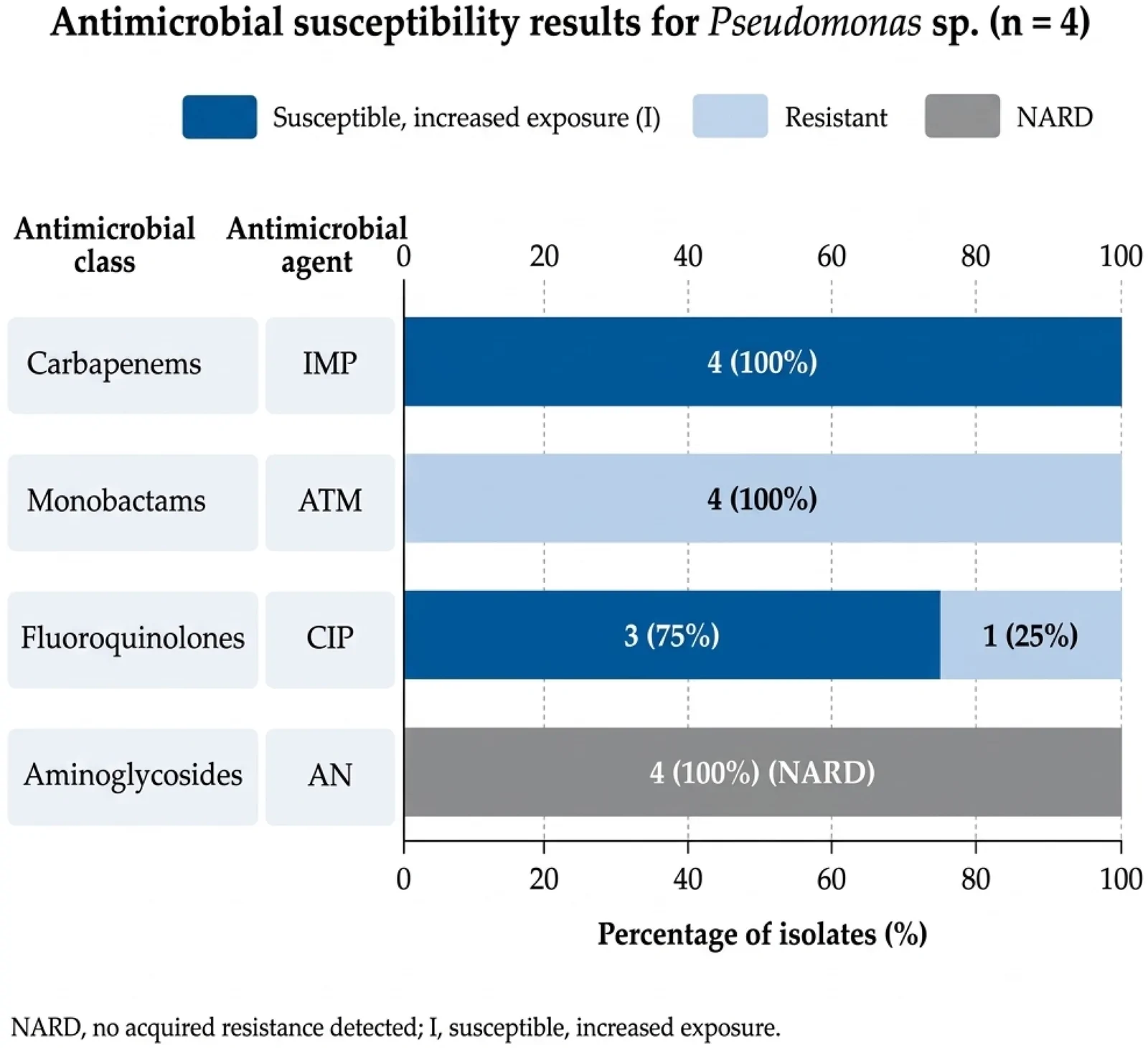
Antimicrobial susceptibility results for *Pseudomonas* sp. (*n* = 4).

Table 3 and Figure 6 present the antimicrobial susceptibility panel tested for the *Pseudomonas* sp. isolates. Among *Pseudomonas* sp. (*n* = 4), no isolate fulfilled the operational MDR definition. Three isolates were resistant to aztreonam only (3/4, 75.0%) and one isolate was resistant to ciprofloxacin plus aztreonam (1/4, 25.0%).

**Figure 7:**
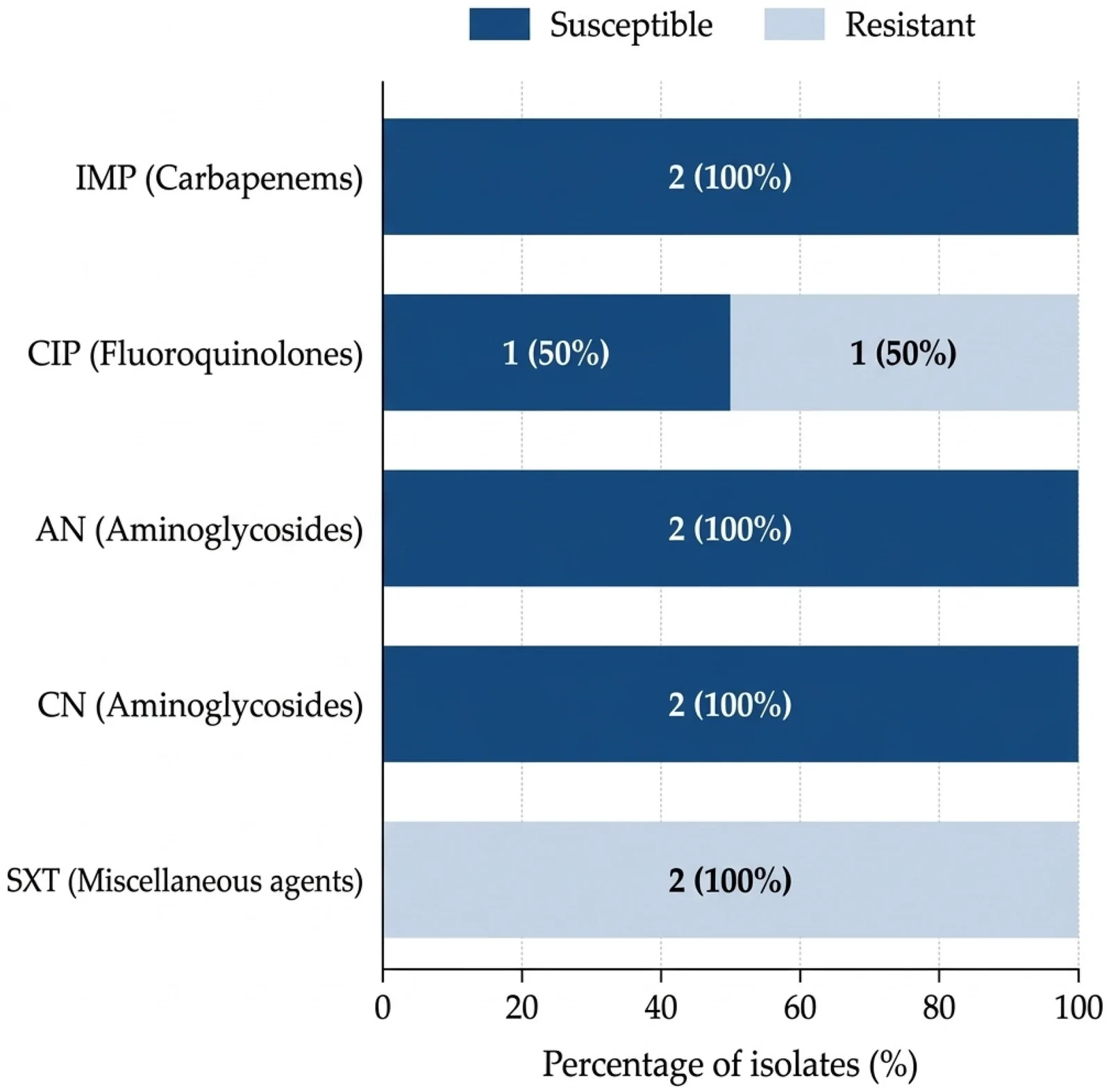
Antimicrobial susceptibility results for *Acinetobacter* sp. (*n* = 2).

Table 4 and Figure 7 present the *Acinetobacter* sp. antimicrobial susceptibility. Results showed that none of the *Acinetobacter* sp. isolates exhibited an MDR phenotype based on the antimicrobial panel tested. Both isolates (2/2; 100%) were resistant to trimethoprim–sulfamethoxazole, while one isolate (1/2; 50%) additionally exhibited resistance to ciprofloxacin. All isolates remained susceptible to imipenem, amikacin and gentamicin. Among *Acinetobacter* sp. (*n* = 2), neither isolate was classified as MDR. One isolate was resistant to trimethoprim–sulfamethoxazole only (1/2, 50.0%) and the other was co-resistant to trimethoprim–sulfamethoxazole and ciprofloxacin (1/2, 50.0%).

The antimicrobial response profiles varied across the 22 *Staphylococcus* spp. Isolates, with the number of resistant antimicrobial classes ranging from 0 to 5. Five isolates (22.7%) showed no resistance to the tested agents, four (18.2%) were resistant to one antimicrobial class, one (4.5%) to two classes, four (18.2%) to three classes, seven (31.8%) to four classes, and one (4.5%) to five classes.

**Figure 8:**
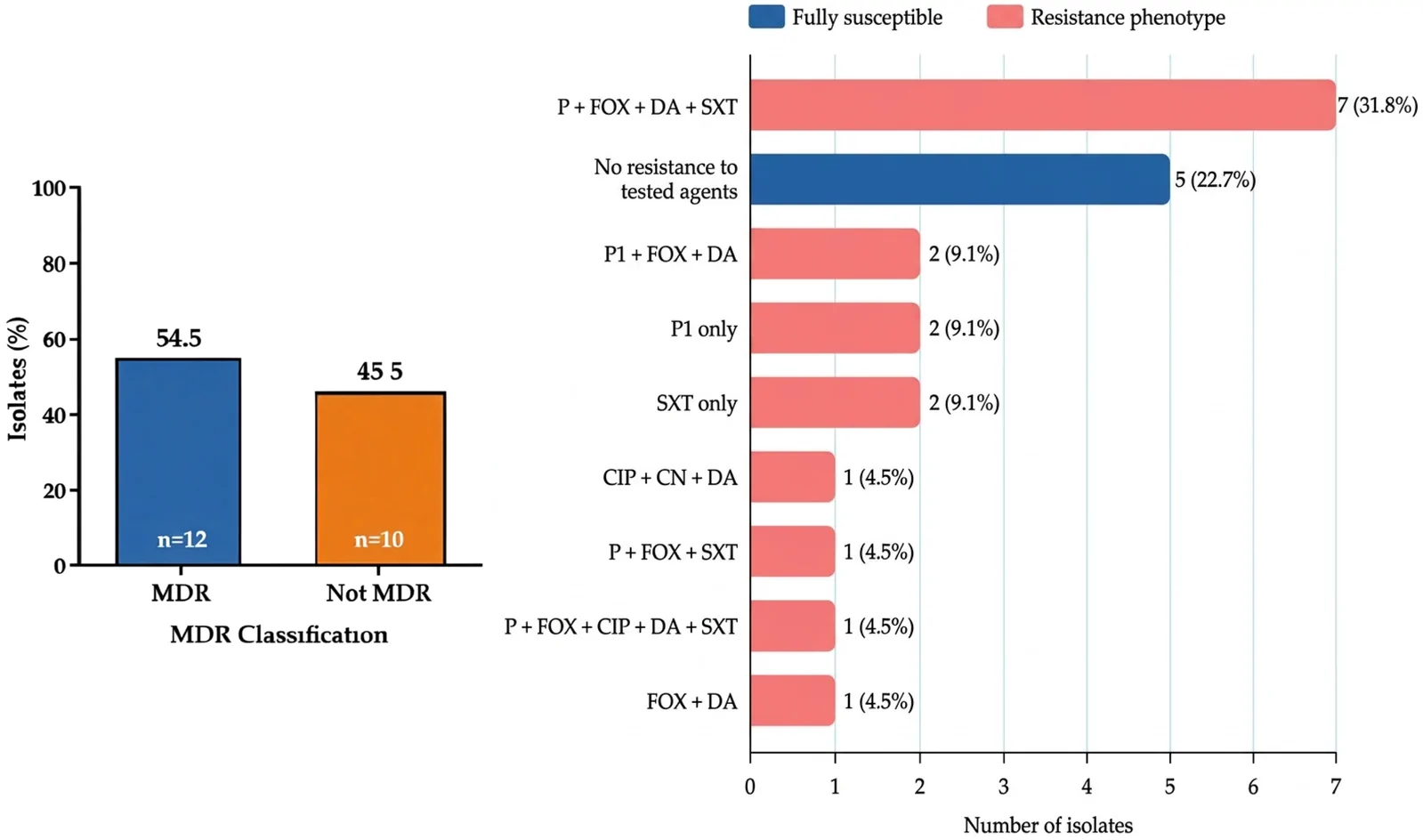
MDR classification results and resistance phenotype results for *Staphylococcus* sp. (*n* = 22).

Figure 8 describes the MDR classification results and resistance phenotype results for *Staphylococcus* sp., showing that, among the 22 *Staphylococcus* sp. isolates, 12 (54.5%) were classified as multidrug-resistant (MDR), whereas 10 isolates (45.5%) did not meet the criteria for MDR classification. Among the *Staphylococcus* sp. isolates, nine distinct antimicrobial susceptibility/resistance profiles were identified. The most frequent resistance pattern was P1 + FOX + DA + SXT, observed in seven isolates (31.8%), while five isolates (22.7%) showed no resistance to the tested agents. Resistance to P1 + FOX + DA, P1 alone, and SXT alone was detected in two isolates each (9.1%). The remaining phenotypes—CIP + CN + DA, P1 + FOX + SXT, P1 + FOX + CIP + DA + SXT, and FOX + DA—were each observed in a single isolate (4.5%). Overall, 17 of the 22 isolates (77.3%) exhibited resistance to at least one of the antimicrobial agents tested.

**Figure 9:**
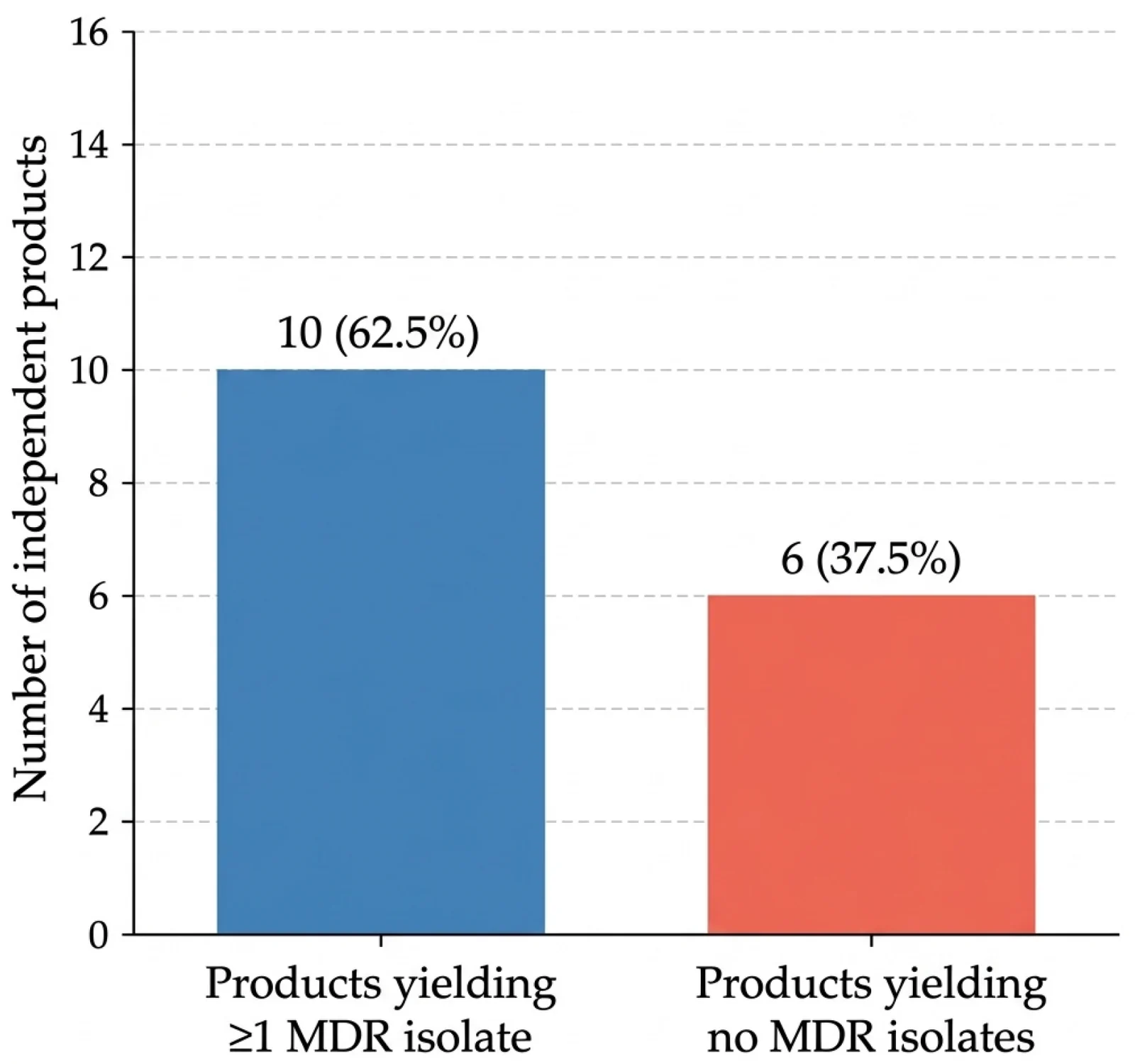
*Staphylococcus* MDR at the product level.

**Figure 10:**
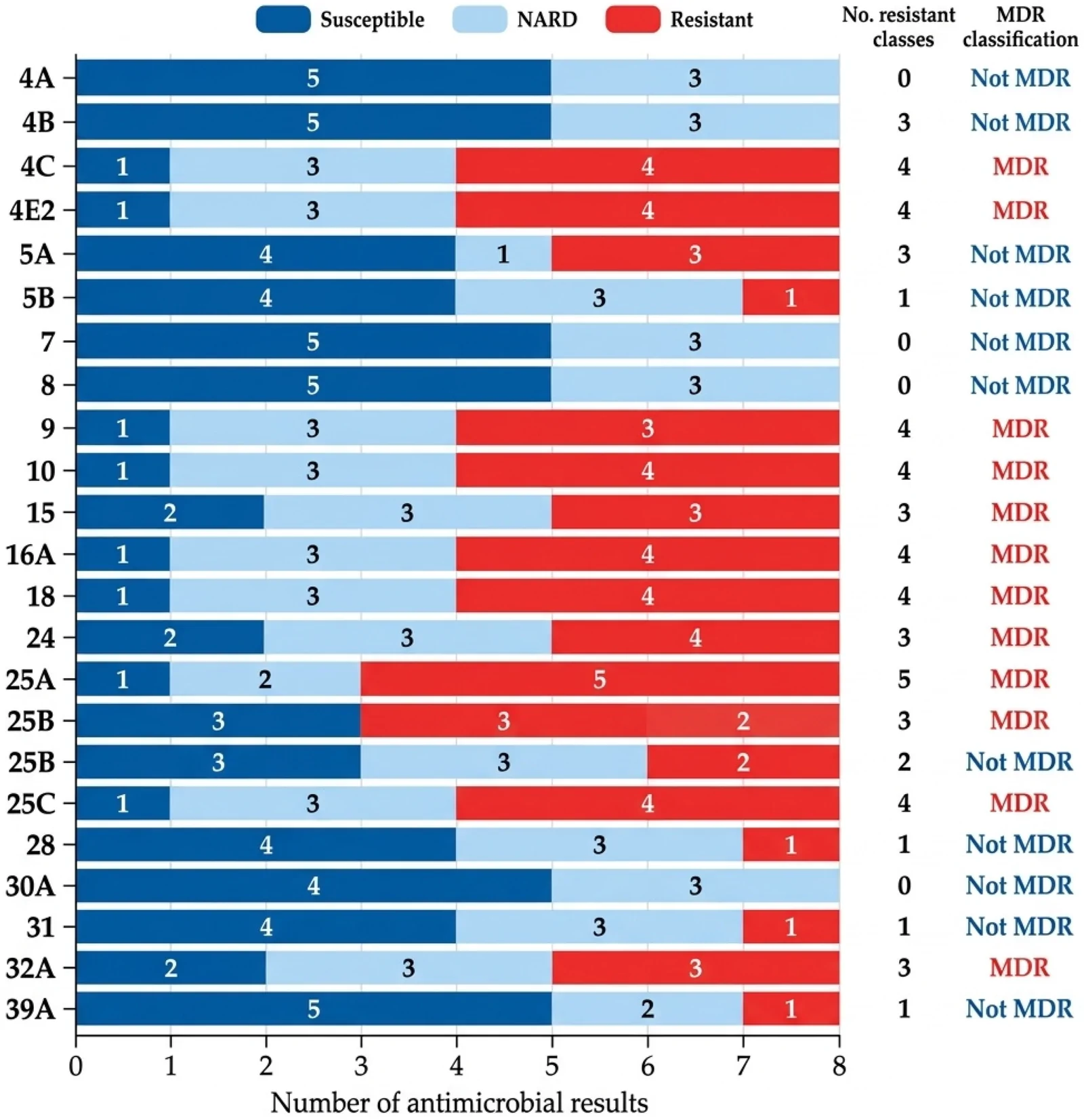
Antimicrobial MDR profile of *Staphylococcus* isolates.

At the product level, among the 16 independent products from which *Staphylococcus* spp. were recovered, 10 (62.5%) yielded at least one isolate classified as MDR (Figure 8 and Figure 9), while overall, at the isolate level, analysis showed that 12 of the 22 isolates (54.5%) met the criteria for MDR classification, whereas 10 (45.5%) were classified as non-MDR (Table 5 and Figure 10).

Among the resistance patterns observed, cefoxitin resistance (used as a surrogate for methicillin resistance) and resistance to macrolides and lincosamides, particularly clindamycin, stand out as clinically relevant, since they could restrict treatment options if these isolates were to cause infection. Other resistances, such as penicillin resistance, are frequent in staphylococci and carry less clinical weight in this setting.

**Figure 11:**
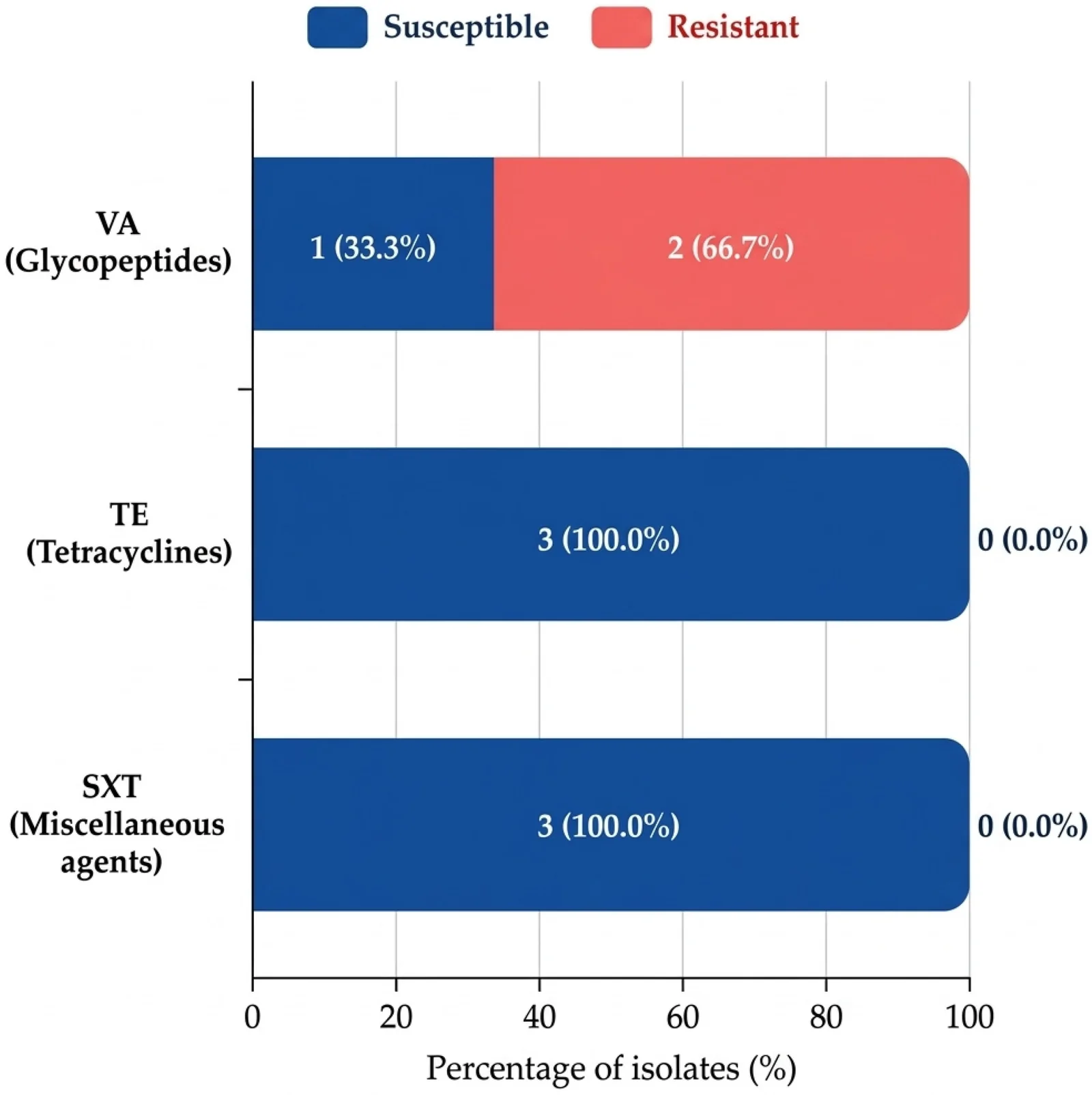
Antimicrobial susceptibility results for *Streptococcus* sp. (*n* = 3).

Among the three *Streptococcus* isolates, none was classified as MDR. Two isolates showed a vancomycin-resistant disk-diffusion phenotype (2/3, 66.7%) and one isolate was susceptible to all agents tested. All three isolates remained susceptible to tetracycline and trimethoprim–sulfamethoxazole (Table 6 and Figure 11).

**Figure 12:**
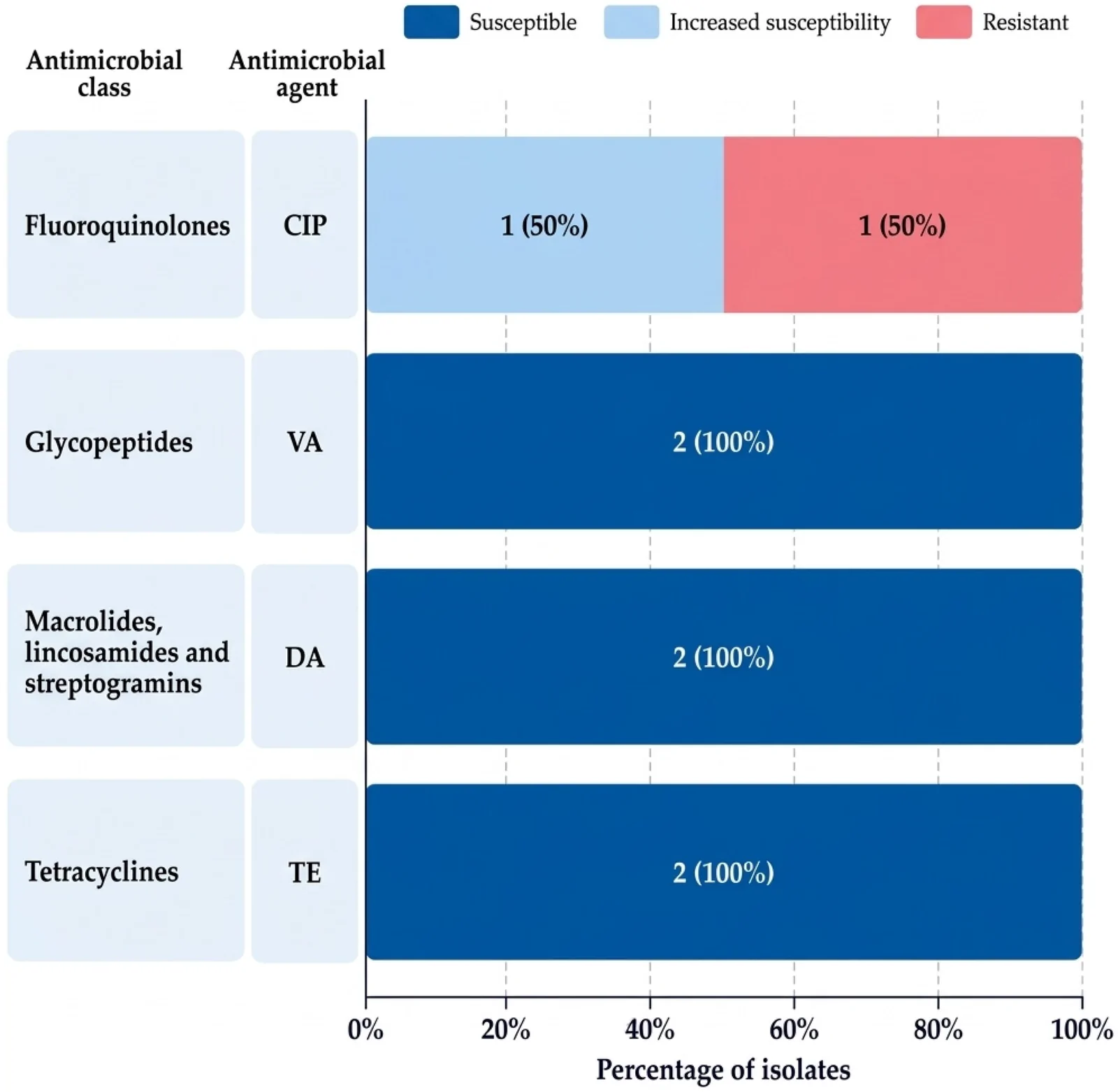
Antimicrobial susceptibility results for *Bacillus* sp. (*n* = 2).

Among the two *Bacillus* isolates, neither was classified as MDR. One isolate was resistant to ciprofloxacin (50%); the second isolate showed increased susceptibility to ciprofloxacin and was otherwise non-resistant (50%). Both isolates remained susceptible to clindamycin and tetracycline (Table 7 and Figure 12).

#### 3.4.2. Resistance Burden by Bacterial Genus

Resistance proportions were summarised descriptively for each bacterial genus using the median and observed range. The proportion of antimicrobial classes where resistance was identified was calculated separately for each isolate. These descriptive results are presented in Table 8. The proportion of antimicrobial classes exhibiting acquired resistance was calculated for each isolate. These values are presented descriptively given the small and unequal numbers of isolates represented within each genus.

**Figure 13:**
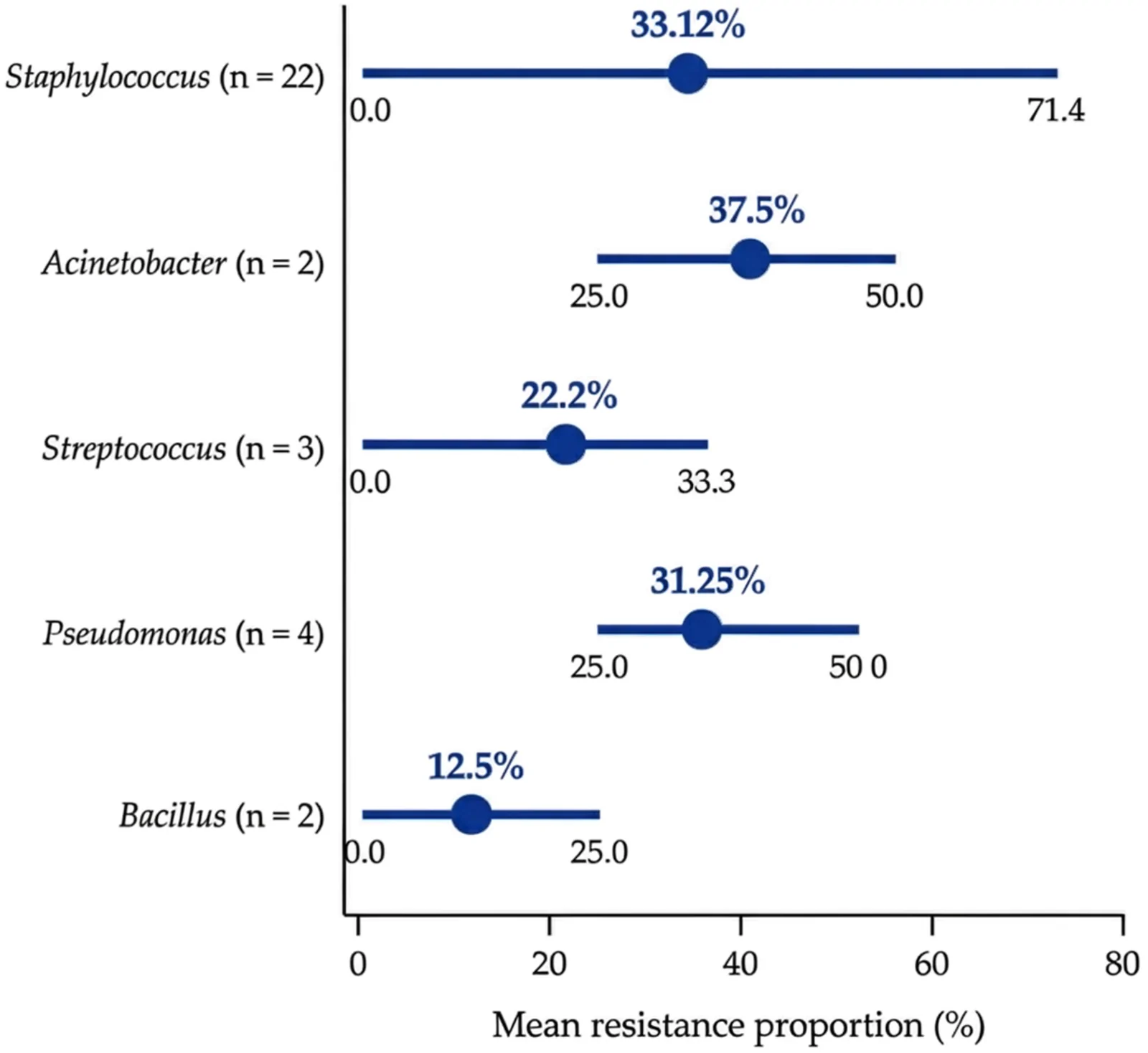
Mean proportion of antimicrobial resistance. Isolate-level proportion of tested antimicrobial classes classified as resistant, by bacterial genus. Points indicate means and horizontal lines indicate observed ranges.

Resistance proportions were summarised descriptively for each bacterial genus as the proportion of antimicrobial classes in which each isolate exhibited resistance to at least one tested agent. Inferential comparisons of resistance proportions between genera were not performed because the antimicrobial panels differed in both the number and composition of antimicrobial classes tested across genera, precluding direct comparability of these proportions. In addition, multiple isolates were recovered from some individual cosmetic products and therefore could not be assumed to represent fully independent observations. Treating all isolates as independent units in an isolate-level between-genus comparison could introduce pseudoreplication and artificially inflate the effective sample size. Accordingly, resistance proportions were reported descriptively as the median and range for each genus. In addition, as a complementary descriptive measure, mean resistance proportions were also calculated for each genus. The mean resistance proportions were 37.5% for *Acinetobacter*, 33.12% for *Staphylococcus*, 31.25% for *Pseudomonas*, 22.2% for *Streptococcus*, and 12.5% for *Bacillus* (Figure 13).

#### 3.4.3. Antibiotic-Specific Resistance by Genus

**Figure 14:**
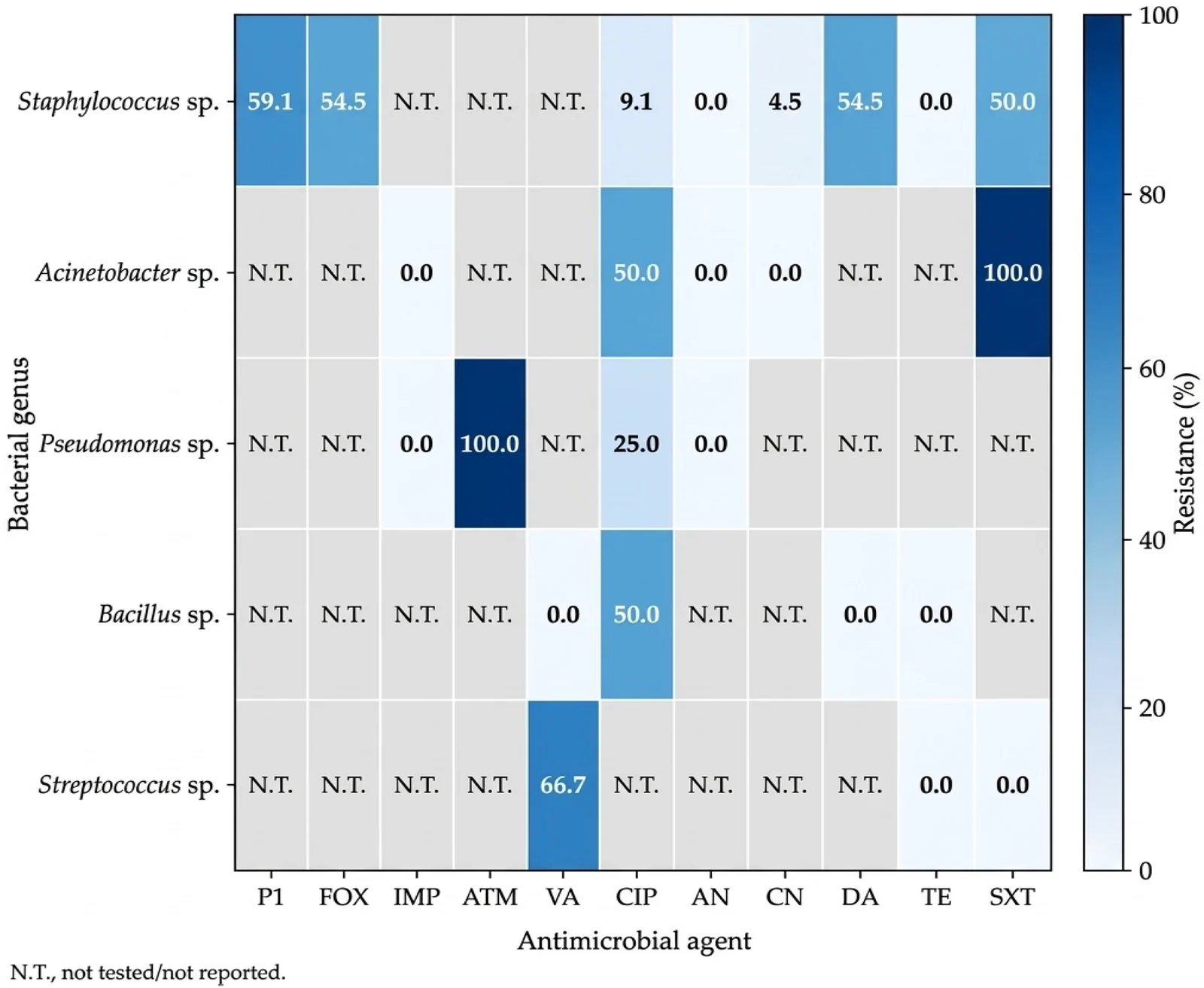
Antibiotic-specific resistance by genus, expressed as the number of resistant isolates divided by the number tested, *n*/N (%).

Penicillin resistance was observed in 59.09% of *Staphylococcus* sp. isolates (*n* = 13/22). Cefoxitin resistance was detected in 54.5% of *Staphylococcus* sp. isolates (*n* = 12/22). All *Acinetobacter* sp. (*n* = 2/2) were susceptible to gentamicin, whereas among *Staphylococcus* isolates, 4.5% (*n* = 1/22) were resistant and 95.5% (*n* = 21/22) were classified as having no acquired resistance detected (NARD). Ciprofloxacin resistance was observed in 50.0% of *Acinetobacter* sp. (*n* = 1/2), 25.0% of *Pseudomonas* sp. (*n* = 1/4), and 9.1% of *Staphylococcus* sp. isolates (*n* = 2/22), while ciprofloxacin resistance was observed in 1/2 (50.0%) Bacillus isolates. Clindamycin resistance was observed in 54.5% of *Staphylococcus* sp. isolates (*n* = 12/22), while both *Bacillus* sp. isolates were susceptible (*n* = 2/2) (Figure 14). No tetracycline-resistant isolates were identified. Trimethoprim–sulfamethoxazole resistance was observed in all *Acinetobacter* sp. isolates (*n* = 2/2; 100%), half of the *Staphylococcus* sp. isolates (*n* = 11/22; 50.0%), and none of the *Streptococcus* sp. isolates (*n* = 0/3; 0%).

#### 3.4.4. Antibiotic Class Resistance by Genus at the Product Level

**Figure 15:**
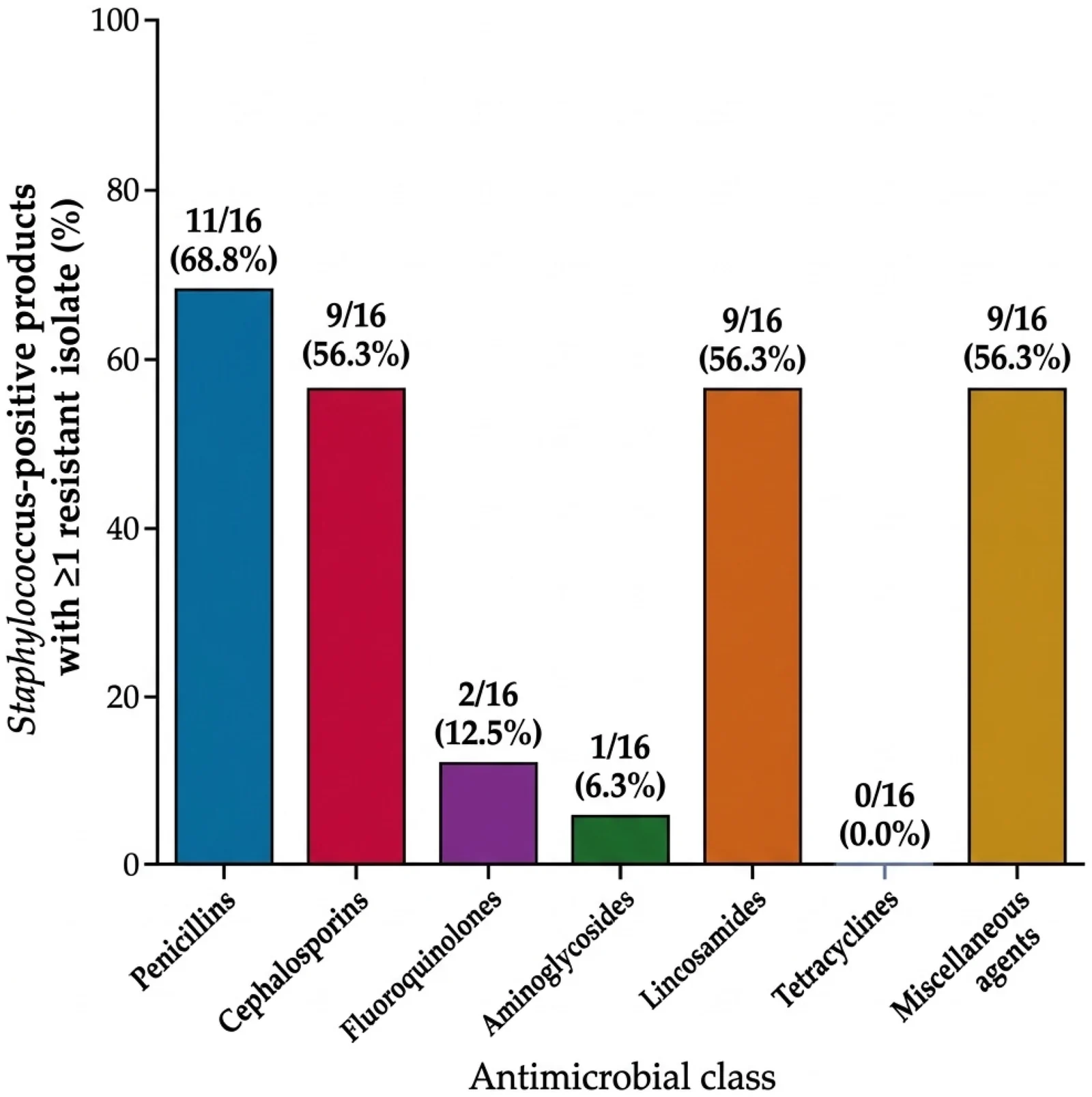
Product-level detection of antimicrobial resistance among *Staphylococcus-*positive cosmetic products. Each bar represents the percentage of independent cosmetic products (*n* = 16) from which at least one *Staphylococcus* sp. isolate exhibited resistance to the respective antimicrobial class. Products yielding multiple *Staphylococcus* sp. isolates were counted once per class.

At the product level, resistance to penicillins was detected in *Staphylococcus* isolates recovered from 11 of the 16 independent *Staphylococcus*-positive products (68.8%). Resistance to cephalosporins, lincosamides and miscellaneous agents was detected in isolates from 9/16 products each (56.3%), whereas resistance to fluoroquinolones and aminoglycosides was detected in 2/16 (12.5%) and 1/16 (6.3%) products, respectively. No tetracycline-resistant *Staphylococcus* isolates were recovered from the 16 products (Figure 15).

#### 3.4.5. Descriptive Distribution by Gram Group

**Figure 16:**
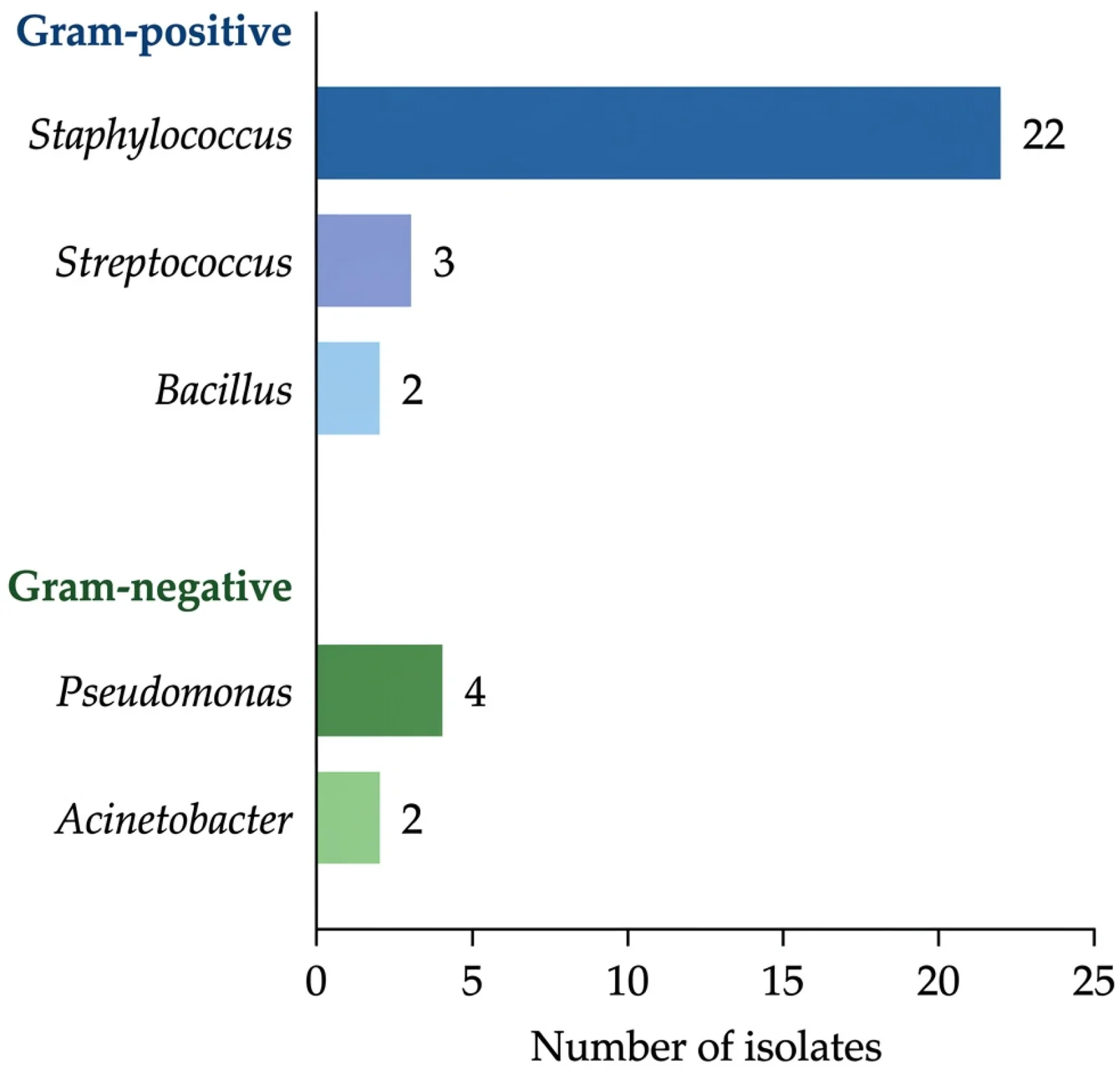
Distribution of isolates by genus and Gram group.

A total of 33 bacterial isolates were included in the antimicrobial susceptibility analysis. Gram-positive bacteria predominated, accounting for 27/33 isolates (81.8%), whereas Gram-negative bacteria represented 6/33 (18.2%). *Staphylococcus* was the most frequently recovered genus, comprising 22/33 isolates (66.7%), followed by *Pseudomonas* (4/33; 12.1%), *Streptococcus* (3/33; 9.1%), *Acinetobacter* (2/33; 6.1%), and *Bacillus* (2/33; 6.1%) (Figure 16).

#### 3.4.6. Beta-Haemolytic Activity

**Figure 17:**
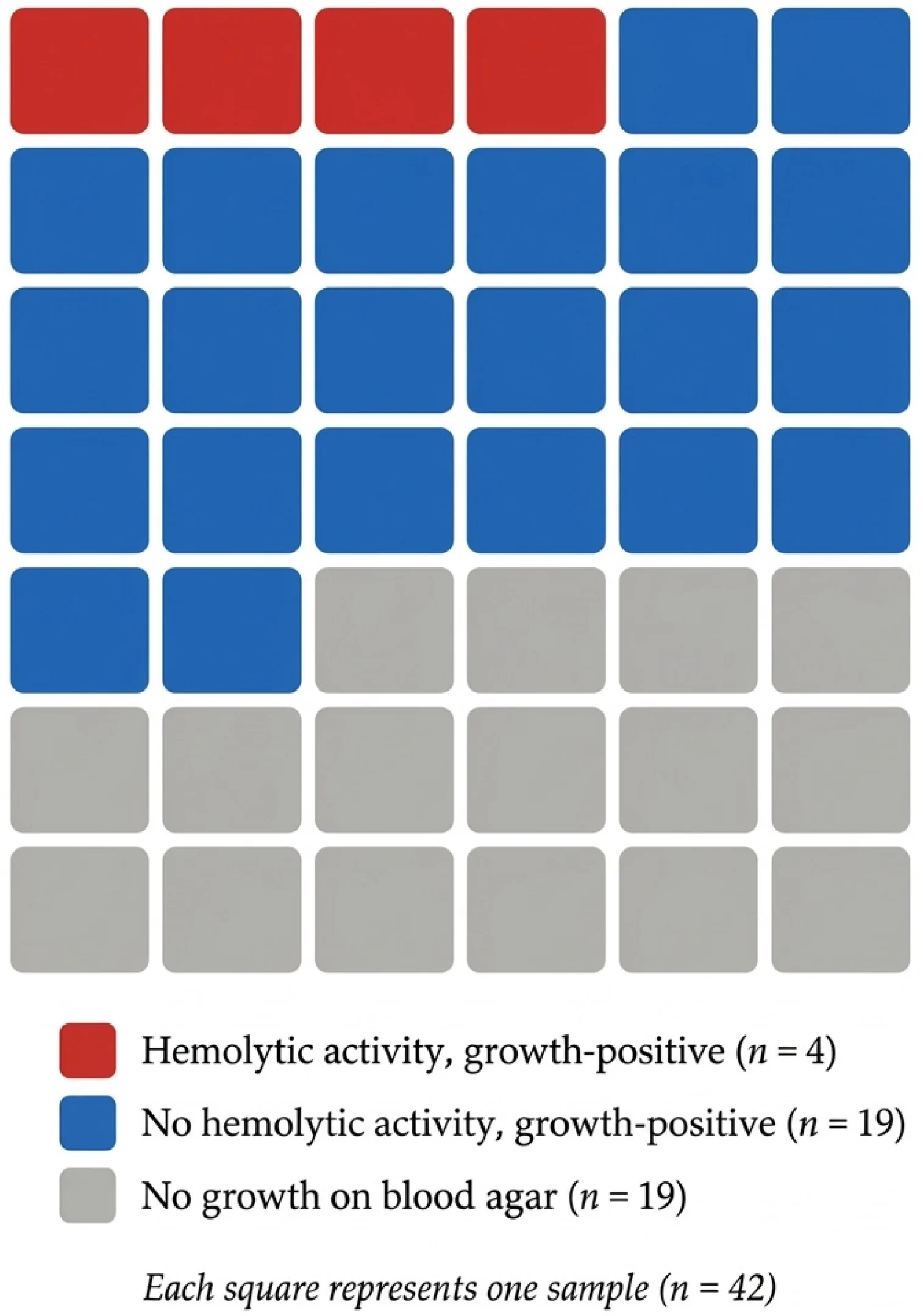
Blood agar growth and beta-haemolytic activity among the analysed cosmetic samples.

Of the 42 samples analysed, 23 (54.8%) showed growth on blood agar (Figure 17). Among these 23 growth-positive samples, four (4/23; 17.4%) yielded isolates exhibiting beta-haemolytic activity. This finding is relevant to the microbiological safety assessment of cosmetic products, in which viable, potentially harmful microorganisms should be absent or adequately controlled.

## 4. Discussion

This study found that more than half of the cosmetic testers sampled from community pharmacies in northern Portugal (25/42; 59.5%) yielded detectable microbial growth under the applied culture conditions, with *Staphylococcus* sp. representing the predominant bacterial genus. In addition, 12 of the 33 characterised bacterial isolates (36.4%) met the study-defined operational multidrug-resistance (MDR) criterion. The observed frequency of microbial recovery falls within the broad range reported for other shared-use and in-store cosmetic products, although direct comparisons between studies should be made cautiously because of differences in product types, sampling strategies, microbiological methods and use environments. This contamination burden sits within the range reported for other shared-use and in-store cosmetic products, although comparisons across studies must be made cautiously given differences in product type, sampling method, and culture protocol. Bashir and Lambert [8] found that 79–90% of used cosmetic products donated by UK consumers (lipsticks, lip glosses, eyeliners, mascaras and beauty blenders) were contaminated with bacteria, including *Staphylococcus aureus* and *Escherichia coli*, while Noor et al. [9] reported bacterial contamination in the majority of unsealed makeup testers sampled from retail outlets in Saudi Arabia, with *Staphylococcus epidermidis* as the most frequently isolated species (57% of isolates), a genus-level pattern consistent with our own findings.

The lower proportion of testers yielding microbial growth here relative to some previous studies may reflect differences in product-category composition, patterns of use, tester turnover, retail handling or microbiological methodology. However, these factors were not measured and therefore cannot be identified as explanations for the observed differences. In addition, because preservative neutralisation was not performed before culture, samples in which no microbial growth was detected cannot be considered free of microorganisms, and residual antimicrobial activity may have reduced microbial recovery under the applied experimental conditions. The culture-based approach may also influence the taxonomic distribution recovered and therefore does not characterise the complete microbial community present in each tester.

A recent study of lipstick testers from retail shops also recovered antibiotic-resistant bacteria, including *Staphylococcus gallinarum* and *Micrococcus luteus,* and recommended single-use applicators and regular tester hygiene [28]. Although the scope and sampling design of that study differed from this investigation, both studies demonstrate that bacteria exhibiting phenotypic antimicrobial resistance can be recovered from cosmetic testers.

The predominance of *Staphylococcus* sp. among the isolates recovered is consistent with its status as the dominant genus of the resident and transient human skin microbiota, and with its recognised role as an opportunistic pathogen once skin barrier integrity is compromised [5]. Repeated finger and applicator contact during in-store testing provides a plausible route for this normally commensal genus to be transferred from consumers’ skin onto the tester itself, and subsequently to other users [29]. This transmission pathway has been documented for cosmetic testers by Bedaida et al. [10], who recovered antibiotic-resistant *Staphylococcus* sp. and *Bacillus* sp. isolates from 325 makeup testers sampled in Algerian beauty retailers. They concluded that testers could act as a reservoir and inferred transmission vehicle for antibiotic-resistant Gram-positive bacteria. These observations support the relevance of repeated consumer handling as a possible contributor to contamination, although contamination introduced during manufacturing, distribution, storage or retail handling cannot be excluded.

The comparatively high proportions of detectable microbial growth observed among anti-ageing products (7/8; 87.5%) and sunscreens (10/16; 62.5%) are also noteworthy, although these findings should be interpreted descriptively because of the small and unequal numbers of testers represented within the different product categories. Differences in formulation and use of characteristics provide plausible hypotheses for these observations. Water activity, product texture, dispensing system, environmental exposure and repeated consumer contact can influence the microbiological stability of cosmetic products [29]. For example, packaging systems that reduce direct exposure of the bulk formulation may provide fewer opportunities for microbial introduction than open or repeatedly accessed containers. Conversely, formulations with conditions less favourable to microbial survival, including lower water availability, may be less permissive to microbial persistence. However, packaging type, applicator design, water activity, tester age, frequency of use and replacement history were not systematically recorded in this study. The higher observed proportions of microbial growth among anti-ageing products and sunscreens therefore cannot be attributed to any of these factors based on the present data and should instead be regarded as hypothesis-generating observations for future investigation.

A notable finding of this study was that all isolates meeting the study-defined operational MDR criterion belonged to the genus *Staphylococcus* sp. Specifically, 12 of 22 *Staphylococcus* sp. isolates (54.5%) met this criterion, whereas none of the 11 isolates belonging to the remaining genera did so. However, this distribution should not be interpreted as evidence that multidrug resistance was intrinsically more frequent in *Staphylococcus* sp. than in the other genera recovered. The genus-specific antimicrobial panels differed in both the number and identity of antimicrobial categories represented, and therefore the opportunity for an isolate to meet the operational MDR criterion was not equivalent across genera. In addition, several isolates originated from the same cosmetic tester, precluding treatment of all isolate-level observations as statistically independent. For these reasons, no inferential comparison of MDR frequency or resistance burden between bacterial genera was retained in the revised analysis. This operational classification should be distinguished from formal application of the organism-specific acquired non-susceptibility definitions proposed by Magiorakos et al. [25]. The pooled MDR proportion is therefore a descriptive result specific to the isolate collection and testing framework used here.

The additional product-level summary counted each *Staphylococcus*-positive tester only once per antimicrobial category. This reduces repeated contributions from the same tester, but products yielding more isolates may still have had a greater opportunity to contain a detected resistance phenotype. These proportions describe the *Staphylococcus*-positive testers and should not be interpreted as resistance prevalence among all sampled products.

Within the *Staphylococcus* sp. group, the isolates meeting the present study’s MDR criterion were predominantly coagulase-negative staphylococci, reflecting the overall species composition of the recovered *Staphylococcus* sp. population, in which *S. epidermidis* and *S. saprophyticus* were more frequent than *S. aureus*. Coagulase-negative staphylococci are common members of the human microbiota but can also act as opportunistic pathogens and reservoirs of antimicrobial-resistance determinants [5]. Among the individual antimicrobial phenotypes observed in the *Staphylococcus* sp. panel, cefoxitin resistance and clindamycin resistance were particularly notable. Cefoxitin is used by EUCAST as a phenotypic screening agent for methicillin resistance in staphylococci, although this study did not include molecular confirmation of the underlying resistance determinants. Similarly, clindamycin resistance was detected by disk diffusion, but inducible clindamycin resistance was not specifically investigated. These findings therefore support the presence of phenotypically detectable antimicrobial resistance among *Staphylococcus* sp. isolates recovered from cosmetic testers, but they should not be interpreted as evidence of specific resistance mechanisms or as direct indicators of clinical outcome. For agents interpreted using EUCAST breakpoints in brackets, the study-defined category “No acquired resistance detected” (NARD) does not imply clinical susceptibility [15,30]. Following review of the applicable EUCAST criteria, vancomycin results were retained only for microorganisms with breakpoints applicable to the testing method used. Metronidazole results were excluded from categorical susceptibility interpretation because no applicable breakpoints were available for the microorganisms tested [15].

The aztreonam-resistant *Pseudomonas* sp. and trimethoprim–sulfamethoxazole-resistant Acinetobacter sp. isolates also warrant further characterisation, although the small numbers recovered and genus-level identification preclude broader conclusions about resistance frequencies or mechanisms. Notably, Pseudomonas is not intrinsically resistant to aztreonam; *P. aeruginosa* nonetheless has a well-documented capacity to acquire resistance to this and other beta-lactams through upregulation of efflux pumps such as MexAB-OprM, loss or downregulation of the OprD porin, and production of chromosomal or acquired beta-lactamases [31]. The aztreonam-resistant phenotype observed here therefore indicates that the recovered strain is no longer wild-type with respect to this agent, which is of interest when considering the clinical relevance of testers as a potential reservoir for such adaptively resistant strains. The vancomycin-resistant classifications retained for Streptococcus sp. require confirmation of identification and susceptibility before broader biological conclusions can be drawn.

Coagulase-negative staphylococci are recognised as a reservoir of mobile antimicrobial-resistance determinants (e.g., the mec and erm gene families) that can be acquired through repeated, low-level antibiotic or biocide exposure and are capable of horizontal transfer to more virulent staphylococcal species, including *S. aureus*; repeated finger/applicator contact and cumulative multi-user handling of an in-store tester provide a plausible route for such resistant skin-commensal populations to accumulate over the tester’s service life. By contrast, the small number of non-*Staphylococcus* isolates recovered here (*n* = 11, spanning four genera) limits the power of this sub-analysis considerably, and the numerically low MDR frequency observed among them in this particular sample should not be read as evidence that *Pseudomonas* sp. or *Acinetobacter* sp. are inherently less resistant in this setting; genus-level comparisons were precluded by the small number of isolates recovered for each non-*Staphylococcus* genus. Confirming whether this *Staphylococcus*-driven MDR pattern reflects genuine biological selection at the tester surface, rather than the small overall isolate numbers in this study, would require genotypic characterisation of resistance determinants, which was outside the scope of this phenotypic disk-diffusion study. Neither preservative susceptibility nor horizontal gene transfer was investigated, and the observed resistance phenotypes do not establish selection or transfer of resistance determinants within the testers.

The recovery of *Bacillus* spp. from cosmetic testers may also be biologically plausible given the ability of members of this genus to form endospores with increased tolerance to a range of environmental and chemical stresses [32,33]. However, only two *Bacillus* isolates were recovered in this study, and no assessment of sporulation, preservative susceptibility or persistence within the cosmetic formulations was performed. Their recovery therefore cannot be attributed to enhanced survival of endospores or to failure of any specific preservative system. Rather, the presence of *Bacillus* spp. should be interpreted as part of the taxonomic diversity of bacteria recoverable from the sampled testers under the applied culture conditions.

The beta-haemolytic phenotype observed on blood agar provides an additional phenotypic characteristic of the recovered microorganisms, but does not by itself establish increased pathogenicity or consumer health risk [34]. Haemolytic determinants were not characterised, and no cytotoxicity, inflammatory-response or infection assays were performed. Consequently, the clinical significance of this phenotype remains undetermined.

The recovery of multiple distinct microorganisms from some individual testers further highlights the distinction between the microbiological quality of a cosmetic formulation before use and its microbiological status after repeated exposure in a shared retail environment. Preservative efficacy is evaluated under standardised laboratory conditions, whereas in-use cosmetic testers may be subjected to repeated consumer contact and environmental exposure over time. However, this study did not assess preservative efficacy, tester age, cumulative frequency of use, replacement history or the persistence of individual microorganisms within the formulations. It is therefore not possible to determine whether polymicrobial recovery resulted from repeated microbial introduction, persistence or proliferation within the product, or other unmeasured factors. Nevertheless, the recovery of multiple microbial taxa from individual testers supports consideration of shared cosmetic testers as a distinct in-use context from unopened retail products and provides a rationale for further investigation of handling, hygiene and replacement practices.

In Portugal, cosmetic distributors and retailers are subject to regulatory requirements governing the distribution and retail provision of cosmetic products; however, the cited national legislation does not itself establish tester-specific standardised procedures defining cleaning or disinfection frequency, replacement intervals, or consumer handling practices [35]. At the manufacturing stage, cosmetic products are subject to defined Good Manufacturing Practice requirements intended to ensure product quality and safety [2]. European market-surveillance data have additionally documented microbiological non-compliance among cosmetic products placed on the market, including cases involving Gram-negative bacteria such as *Pseudomonas* spp. [14]. Accordingly, the present findings do not allow for the attribution of the contamination detected in these testers specifically to consumer handling.

From a public and One Health perspective, these findings reinforce that community pharmacy cosmetic testers should not be regarded as a negligible AMR exposure route. Although the concentrations and exposure durations involved in tester use are far lower than in clinical settings, both the WHO Bacterial Priority Pathogens List [21] and the broader antimicrobial-resistance literature emphasise that community and environmental reservoirs, not only hospitals, contribute to the overall burden and dissemination of resistant organisms. Cosmetic testers, as a point of repeated, unsupervised, multi-user contact in a healthcare-adjacent retail setting (the community pharmacy), occupy a distinctive niche in this landscape that, as Bedaida et al. (2025) also argue, remains comparatively understudied relative to other shared-use consumer products [10]. The potential clinical relevance of the present findings lies in the recovery of viable bacteria exhibiting phenotypic antimicrobial resistance from cosmetic testers intended for repeated use by multiple consumers. These observations support further investigation of microbial transfer during tester use and of handling, hygiene and replacement practices that may influence microbial contamination.

The limitations of this study include the sampling of testers from a region of Portugal (north of the country) over a two-month period, which limits generalisation to other regions, seasons, or pharmacy types; the relatively small number of testers within several product categories, which restricts the power of category-level comparisons to an exploratory role (as already noted); the absence of data on tester age, replacement frequency, and in-store hygiene practices, all plausible contributors to contamination that could not be assessed from the present study design; and the use of phenotypic susceptibility testing alone, without molecular confirmation of the resistance mechanisms or genes underlying the observed MDR phenotypes. In addition, samples were plated directly, without an additional preservative-neutralisation step. While this is consistent with common practice for assessing testers already in active use, it is possible that residual antimicrobial activity from the product itself moderated recovery in some samples, meaning that the reported contamination rate (59.5%) may represent a conservative rather than absolute estimate of prevalence [8,9,10,13]. In addition, the D-zone test for inducible clindamycin resistance, recommended for *Staphylococcus* sp., *Sreptococcus* sp. and beta-haemolytic *Streptococcus* sp., was not performed in this study; isolates reported as clindamycin-susceptible could therefore include unrecognised inducible resistance, which represents a further limitation of the susceptibility data presented here. Finally, the limitations also include the fact that the antimicrobial panel did not encompass all antimicrobial categories included in the EUCAST guidelines for susceptibility testing. Therefore, isolates classified as non-MDR based on the tested panel may have exhibited resistance to additional untested antimicrobial classes. Accordingly, the observed frequency of MDR should be regarded as a conservative panel-dependent estimate and the true prevalence of multidrug resistance may have been underestimated. Importantly, the clinical evidence discussed here is intended to contextualise the potential relevance of the antimicrobial resistance phenotypes detected among cosmetic-derived isolates. The present findings demonstrate the occurrence of antimicrobial-resistant bacteria in cosmetic products but do not establish transmission from cosmetics, subsequent infection, or adverse clinical outcomes.

## 5. Conclusions

This exploratory pilot study demonstrates that viable microorganisms, including bacteria exhibiting phenotypic antimicrobial resistance, can be recovered from in-use cosmetic testers available in community pharmacies in northern Portugal. Microbial growth was detected in 25/42 testers (59.5%), and *Staphylococcus* spp. represented the predominant bacterial genus among the characterised isolates. Phenotypic resistance was detected across several bacterial genera, and 12/33 characterised isolates (36.4%) met the study-defined operational MDR criterion, all within the *Staphylococcus*-specific testing framework. Because antimicrobial panels differed between genera, this finding should be interpreted as a panel-dependent descriptive result rather than as a standardised estimate of MDR prevalence or as evidence that multidrug resistance was intrinsically more frequent in *Staphylococcus* sp.

Resistance phenotypes of potential microbiological relevance were also observed among *Pseudomonas*, *Acinetobacter* and *Streptococcus* spp., including aztreonam resistance in the recovered *Pseudomonas* isolates, trimethoprim–sulfamethoxazole resistance in the *Acinetobacter* isolates, and preliminary vancomycin-resistant phenotypes among *Streptococcus* spp. These findings warrant confirmation through species-level identification and molecular characterisation but do not establish the underlying resistance mechanisms or their clinical consequences. Beta-haemolytic isolates were also recovered from 4/23 blood-agar-positive testers; however, this phenotype should be interpreted as an observed microbiological characteristic rather than as direct evidence of pathogenicity or increased consumer health risk.

Overall, the findings identify in-use cosmetic testers as an understudied non-clinical setting in which antimicrobial-resistant bacteria may be encountered and support further investigation of microbiological surveillance, hygiene and replacement practices for shared-use testers. It is, however, important to note that this study does not demonstrate transmission of microorganisms or resistance determinants to consumers, subsequent colonisation or infection, or associated adverse clinical outcomes. Larger and more standardised studies incorporating preservative neutralisation, broader and harmonised antimicrobial testing, species-level identification, and molecular characterisation will be required to determine the reproducibility, biological basis and potential public-health significance of these observations.

## Figures and Tables

**Table 1:** Microbiological Acceptance Criteria for Finished Cosmetic Products.

Product Type/Application	Acceptable Microbiological Limit	Notes
Topical cosmetic products	≤1 × 10^3^ CFU/g or mL	General limit considered safe for finished cosmetic products.
Products intended for the eye area	≤1 × 10^2^ CFU/g or mL	Require stricter microbiological control.
Products for children under 3 years of age	≤1 × 10^2^ CFU/g or mL	Higher safety requirements due to increased user sensitivity.
Products intended for mucous membranes	≤1 × 10^2^ CFU/g or mL	Includes lips and intimate areas.
All cosmetic products	Absence in 1 g or 1 mL	*Escherichia coli*, *Staphylococcus aureus*, *Pseudomonas aeruginosa*, and *Candida albicans* must be absent.

**Table 2:** Identification of the isolates.

ID	Microorganism
1	*Pseudomonas* sp.
2A	*Acinetobacter* sp.
3	unknown
4A	*Staphylococcus aureus*
4B	*Staphylococcus epidermidis*
4C	*Staphylococcus epidermidis*
4 E1	*Streptococcus* sp.
4 E2	*Staphylococcus saprophyticus*
5A	*Staphylococcus epidermidis*
5B	*Staphylococcus saprophyticus*
6	*Pseudomonas* sp.
7	*Staphylococcus epidermidis*
8	*Staphylococcus epidermidis*
9	*Staphylococcus saprophyticus*
10	*Staphylococcus saprophyticus*
15	*Staphylococcus epidermidis*
16A	*Staphylococcus saprophyticus*
18	*Staphylococcus saprophyticus*
20A	*Streptococcus* sp.
20B	*Bacillus* sp.
24	*Staphylococcus epidermidis*
25A	*Staphylococcus epidermidis*
25B	*Staphylococcus epidermidis*
25C	*Staphylococcus saprophyticus*
28	*Staphylococcus epidermidis*
29	*Streptococcus* sp.
30A	*Staphylococcus epidermidis*
30B	unknown
31	*Staphylococcus epidermidis*
32A	*Staphylococcus saprophyticus*
34	*Pseudomonas* sp.
38	unknown
39A	*Staphylococcus aureus*
39B	*Pseudomonas* sp.
39C	*Bacillus* sp.
40A	unknown
40B	*Acinetobacter* sp.
40C	unknown
40D	unknown

Note: Sample ID: the numeral identifies the tester unit; a letter suffix (A, B, C…) denotes a distinct isolate recovered from the same tester (polymicrobial sample).

**Table 3:** Antimicrobial susceptibility results for *Pseudomonas* sp.

Sample ID	Carbapenems	Monobactams	Fluoroquinolones	Aminoglycosides	No. Resistant Antimicrobial Classes	MDR Classification
	IMP	ATM	CIP	AN		
1	I	R	I	NARD *	1	Not MDR
6	I	R	I	NARD *	1	Not MDR
34	I	R	I	NARD *	1	Not MDR
39B	I	R	R	NARD *	2	Not MDR

I = Susceptible, increased exposure; R = Resistant; NARD = No acquired resistance; IMP, Imipenem; AN, Amikacin; CIP, Ciprofloxacin; ATM, Aztreonam; * For amikacin, EUCAST bracketed breakpoints were applied. Isolates above the resistance threshold were considered to show no phenotypically detectable acquired resistance; classification as S or I was avoided in accordance with EUCAST guidance.

**Table 4:** Antimicrobial susceptibility results for *Acinetobacter* sp. (*n* = 2).

Sample ID	Carbapenems	Fluoroquinolones	Aminoglycosides		Miscellaneous Agents	No. Resistant Antimicrobial Classes	MDR Classification
	IMP	CIP	AN	CN	STX		
2A	S	S	S	S	R	1	Not MDR
40B	S	R	S	S	R	2	Not MDR

**Table 5:** Antimicrobial susceptibility results for *Staphylococcus* sp.

ID	Penicillins	Cephalosporins	Fluoroquinolones	Aminoglycosides		Lincosamides	Tetracyclins	Miscellaneous Agents	No. Resistant Antimicrobial Classes	MDR Classification
	P1	FOX	CIP	AN	CN	DA	TE	STX		
4A	S	S	NARD	NARD	NARD	S	S	S	0	Not MDR
4B	S	S	NARD	NARD	NARD	S	S	S	0	Not MDR
4C	R	R	NARD	NARD	NARD	R	S	R	4	MDR
4E2	R	R	NARD	NARD	NARD	R	S	R	4	MDR
5A	S	S	R	NARD	R	R	S	S	3	MDR
5B	S	S	NARD	NARD	NARD	S	S	R	1	Not MDR
7	S	S	NARD	NARD	NARD	S	S	S	0	Not MDR
8	S	S	NARD	NARD	NARD	S	S	S	0	Not MDR
9	R	R	NARD	NARD	NARD	R	S	R	4	MDR
10	R	R	NARD	NARD	NARD	R	S	R	4	MDR
15	R	R	NARD	NARD	NARD	R	S	S	3	MDR
16A	R	R	NARD	NARD	NARD	R	S	R	4	MDR
18	R	R	NARD	NARD	NARD	R	S	R	4	MDR
24	R	R	NARD	NARD	NARD	S	S	R	3	MDR
25A	R	R	R	NARD	NARD	R	S	R	5	MDR
25B	S	R	NARD	NARD	NARD	R	S	S	2	Not MDR
25C	R	R	NARD	NARD	NARD	R	S	R	4	MDR
28	S	S	NARD	NARD	NARD	S	S	R	1	Not MDR
30A	S	S	NARD	NARD	NARD	S	S	S	0	Not MDR
31	R	S	NARD	NARD	NARD	S	S	S	1	Not MDR
32A	R	R	NARD	NARD	NARD	R	S	S	3	MDR
39A	R	S	NARD	NARD	NARD	S	S	S	1	Not MDR

**Table 6:** Antimicrobial susceptibility results for *Streptococcus* sp.

ID	Glycopeptides	Tetracyclins	Miscellaneous Agents	No. Resistant Antimicrobial Classes	MDR Classification
	VA	TE	STX		
4E1	R	S	S	1	Not MDR
20A	S	S	S	0	Not MDR
29	R	S	S	1	Not MDR

**Table 7:** Antimicrobial susceptibility results for *Bacillus* sp.

Samples ID	Fluoroquinolones	Glycopeptides	Macrolides, Lincosamides and Strepgramins	Tetracyclins	No. Resistant Antimicrobial Classes	MDR Classification
	CIP	VA	DA	TE		
20B	R	S	S	S	1	Not MDR
39C	I	S	S	S	0	Not MDR

**Table 8:** Median proportion of antimicrobial resistance was observed among isolates at the genus level.

Bacterial Genus	*n*	Antimicrobial Classes Tested	Median Resistance Proportion (Range)
*Acinetobacter*	2	4	37.5% (25.0–50.0%)
*Bacillus*	2	4	12.5%(0.0–25.0%)
*Pseudomonas*	4	4	25.0 (25.0–50%)
*Staphylococcus*	22	7	42.9% (0.0–71.4%)
*Streptococcus*	3	3	33.3%(0.0–33.3%)

## Data Availability

The original contributions presented in this study are included in the article. Further inquiries can be directed to the corresponding author.
